# Cellular and Transcriptional Dynamics during Brown Adipose Tissue Regeneration under Acute Injury

**DOI:** 10.34133/research.0268

**Published:** 2023-11-08

**Authors:** Wenjing You, Ziye Xu, Wentao Chen, Xin Yang, Shiqi Liu, Liyi Wang, Yuang Tu, Yanbing Zhou, Teresa G. Valencak, Yizhen Wang, Shihuan Kuang, Tizhong Shan

**Affiliations:** ^1^ College of Animal Sciences, Zhejiang University, Hangzhou, China.; ^2^ The Key Laboratory of Molecular Animal Nutrition, Ministry of Education, Hangzhou, China.; ^3^ Zhejiang Provincial Laboratory of Feed and Animal Nutrition, Hangzhou, China.; ^4^ Department of Laboratory Medicine, the First Affiliated Hospital, College of Medicine, Zhejiang University, Hangzhou, China.; ^5^ Department of Animal Sciences, Purdue University, West Lafayette, IN, USA.

## Abstract

Brown adipose tissue (BAT) is the major site of non-shivering thermogenesis and crucial for systemic metabolism. Under chronic cold exposures and high-fat diet challenges, BAT undergoes robust remodeling to adapt to physiological demands. However, whether and how BAT regenerates after acute injuries are poorly understood. Here, we established a novel BAT injury and regeneration model (BAT-IR) in mice and performed single-cell RNA sequencing (scRNA-seq) and bulk RNA-seq to determine cellular and transcriptomic dynamics during BAT-IR. We further defined distinct fibro-adipogenic and myeloid progenitor populations contributing to BAT regeneration. Cell trajectory and gene expression analyses uncovered the involvement of MAPK, Wnt, and Hedgehog (Hh) signaling pathways in BAT regeneration. We confirmed the role of Hh signaling in BAT development through *Myf5^Cre^-*mediated conditional knockout (cKO) of the *Sufu* gene to activate Hh signaling in BAT and muscle progenitors. Our BAT-IR model therefore provides a paradigm to identify conserved cellular and molecular mechanisms underlying BAT development and remodeling.

## Introduction

The prevalence of obesity worldwide has led to an increase in the risk of metabolic disease and premature death related to lifestyle factors [1]. Brown adipose tissue (BAT) is a thermogenic organ expressing uncoupling protein 1 (UCP1) on the inner mitochondrial membrane, and is believed to be crucial in regulating whole body energy balance [2*–*4]. Brown adipocytes (BAs) act as an efficient energy sink, and effectively burn and dispose of excess fat and glucose when activated [5*–*7]. However, BAT mass and activity decrease during aging [8] and diet-induced obesity [9]. Therefore, there is an urgent need to comprehend the cellular and molecular mechanisms that underlie adult BAT maintenance and regeneration.

Classical BAs are derived from a population of progenitor cells predominantly expressing myogenic factor 5 (*Myf5*) [10*–*12]. During embryogenesis, these progenitor cells invade BAT preadipocytes and then differentiate into mature BAs [13]. Several transcriptional factors have shown to regulate BAT development. *PRDM16* is a crucial factor that drives BA differentiation and controls a brown fat/skeletal muscle switch [10]. *EBF2* determines brown adipocyte identity and regulates brown fat adipogenesis [14*–*16]. *Ewing Sarcoma (EWS)* and *YBX1* regulate BAT development by stimulating *BMP7* production [4*,*^17^]. Recent studies have shown that adult BAT can dynamically remodel to dietary alterations and environmental stimuli [18*,*^19^]. The platelet-derived growth factor receptor alpha (*Pdgfra*) positive adipocyte progenitor cells (*Sca1^+^*/*Pdgfra^+^*) can generate new BAs under cold stress [20]. However, the cellular and molecular mechanisms on adult BAT injury and repair are completely unknown. Some existing BAT removal models do not explicitly reflect the cellular dynamics of BAT development [21*,*^22^]. Therefore, a powerful injury and repair model of BAT is warranted to better understand the cellular and transcriptional dynamics during adult BAT maintenance and regeneration.

Here, we report an acute mouse BAT injury and regeneration model (BAT-IR) by BAT-IR-inducing agent (BAT-IR-IA) injection. Single-cell RNA sequencing (scRNA-seq) and lineage tracing were performed to identify cellular origin and dynamics during BAT injury and repair. We found that a subset of fibro-adipogenic progenitors (FAPs) and myeloid-derived cells contribute to BAT repair and formation. Using RNA sequencing (RNA-seq) and functional genomic approaches, we identified the regulatory pathways underlying BAT regeneration and validated the role of Hedgehog (Hh) signaling in BAT regeneration and development using *Myf5-Cre*-mediated conditional knockout (cKO) of the suppressor of fused (*Sufu*). The finding offers new perspectives in comprehending the cellular and molecular characteristics of adult BAT regeneration. Our study is useful for maintaining BAT functions to combat obesity, diabetes, and other metabolic-related diseases.

## Results

### Construction of an adult BAT injury model (BAT-IR)

**Fig. 1.**
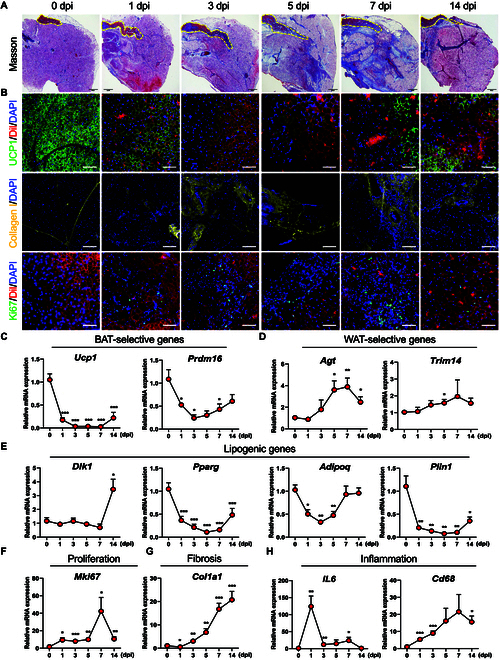
Adult BAT undergoing regeneration during BAT-IR-IA-induced injury. (A) Cross-sectioning and Masson Trichrome staining of BAT at the indicated time points during BAT regeneration. Scale bar: 200 μm. (B) Immunofluorescence of BAT for UCP1 (green), Collagen I (yellow), Ki67 (green), Dil (red), and DAPI (blue) at the indicated time points after BAT-IR-IA injury. Scale bar: 50 μm. (C to H) Relative mRNA levels of BAT-selective (C), WAT-selective genes (D), lipogenic (E), proliferation (F), fibrosis (G), and inflammation-related genes (H) were obtained from the whole BAT with BAT-IR-IA injury. *n* = 6. Error bars represent SEM, *P < 0.05, **P < 0.01, ***P < 0.001, 2-tailed Student’s *t*-test.

To characterize the cellular origin and dynamics during adult BAT remodeling, we generated a model of acute adult BAT injury (BAT-IR) induced by BAT-IR-IA (5% phosphatidylcholine and 5% sodium deoxycholate) injection. Specifically, we chose 0, 1, 3, 5, 7, and 14 days post injury (dpi) to determine immediate immune responses and histological changes by hematoxylin and eosin (H&E) staining and Masson staining (Fig. 1A and Fig. S1A). We found that the homogeneous distribution of multilocular BAs was dramatically disrupted, accompanied by BA necrosis and inflammation at 1 dpi (Fig. 1A and Fig. S1A). At 3 dpi, BAT began to exhibit fibrotic appearance with enlarged spaces and massive interstitial cells infiltrated (Fig. 1A and Fig. S1A). BAs varied in form and cell size; many contained only one large lipid droplet, resembling white adipocytes (Fig. 1A and Fig. S1A). From 3 dpi to 7 dpi, the fibrosis gradually became more severe and the gaps were filled with enlarged adipocytes, each containing a single large lipid droplet (Fig. 1A and Fig. S1A). At 14 dpi, fibrosis in BAT was reduced and the morphology was largely recovered, but the adipocytes were larger than those at 0 dpi (Fig. 1A and Fig. S1A).

Consistent with the histological changes, immunofluorescence (IF) staining revealed that UCP1 levels were downregulated in response to BAT injury, accompanied by increased deposition of type I collagen (accumulation of fibroblasts) and a progressive increase in Ki67^+^ proliferating cells (Fig. 1B). Moreover, we examined the newly generated adipocytes by EDU^+^ labeling and found numerous Edu^+^ adipocytes in BAT-IR-IA-injured BAT at 14 dpi (Fig. S1B and C), indicating new BA formation and BAT regeneration in the BAT-IR model.

We also used real-time qPCR to determine the temporal expression pattern of genes related to BAT, WAT, lipogenesis, proliferation, fibrosis, and inflammation during BAT-IR (Fig. 1C to H and Fig. S1D to F). The BAT-selective genes (e.g., *Ucp1*, *Prdm16, Ebf2*, *Pgc1a*, *Cox5b*, and *Cox7a*) and lipogenic-related genes (e.g., *Pparg*, *Adipoq*, and *Plin1*) were markedly downregulated from 0 dpi to 3 dpi, and increased from 5 dpi to 14 dpi (Fig. 1C and Fig. S1D), reflecting the injury and recovery of BAT. The damaged BAT gradually regenerated and underwent cell proliferation and inflammation. Notably, the WAT-selective genes (e.g., *Agt* and *Trim14*), the proliferation-related gene (e.g., *Mki67*), the fibrosis-related genes (e.g., *Col1a1* and *Col3a1*), and the inflammation-related genes (e.g., *CD68*, *CD86*) were greatly upregulated from 0 dpi to 7 dpi, and reduced from 7 dpi to 14 dpi (Fig. 1D to H and Fig. S1E and F), suggesting that both proliferation and inflammation were more abundant in the initial stage of BAT injury. These results validated our BAT-IR model.

In metabolic cage studies, there were no apparent differences between control and BAT-IR mice in O_2_ consumption, CO_2_ production, heat production, food consumption, and general activity (Fig. S2C to F).

### scRNA-seq resolves cellular heterogeneity during BAT regeneration

**Fig. 2.**
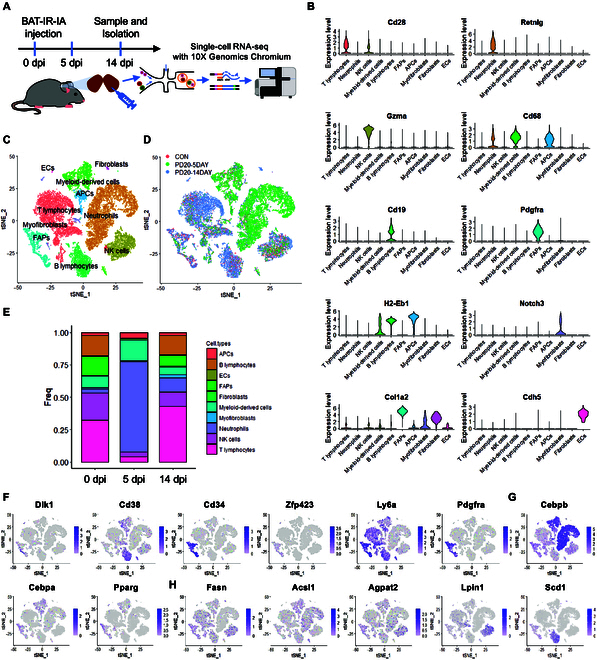
scRNA-seq identified distinct cell populations in BAT-IR-IA-injured BAT. (A) Schematic workflows for BAT scRNA-seq at 0, 5, and 14 dpi. (B) Violin plots grouped by meta clusters demonstrating the expression of cell-type marker genes used to classify the meta clusters. (C) Graph clustering of isolated single cells identifies distinct clusters corresponding to different cell populations. (D) The t-SNE plot of merged isolated single cells from non-injured (0 dpi) and BAT-IR-IA-injured (5 dpi and 14 dpi) BAT. (E) Relative proportions of cell types at each time point. Regeneration time points are plotted along the *x*-axis, and relative abundance as a percentage of total cells is plotted along the *y*-axis. Abbreviations: APCs, antigen-presenting cells; ECs, endothelial cells; FAPs, fibro/adipogenic progenitor cells; NK cells, natural killer cells; dpi, days post-injury. (F to H) Expression of preadipocyte-enriched genes (*Dlk1*, *Cd38*, *Cd34*, *Zfp423*, *Ly6a*, and *Pdgfra*), adipogenic master regulators (*Cebpa*, *Cebpb*, and *Pparg*), and adipogenesis-associated genes (*Fasn*, *Acsl1*, *Agpat2*, *Lpin1*, and *Scd1*) in these populations.

To investigate cellular heterogeneity during BAT repair, we conducted scRNA-seq of cells isolated from the pure BAT of uninjured (0 dpi) and BAT-IR-IA-injured (5 and 14 dpi) mice (Fig. 2A). The normalized scRNA-seq data were analyzed using unsupervised graph-based clustering to identify all cell types. The cell types were then projected onto the t‐distributed stochastic neighbor embedding (t-SNE) plots using the Seurat package in R. Using known markers (Fig. 2B), we classified 10 major cell types, such as FAPs, myeloid-derived cells, B lymphocytes, neutrophils, T lymphocytes, natural killer cells (NK cells), myofibroblasts, antigen-presenting cells (APCs), fibroblasts, and endothelial cells (ECs) (Fig. 2C).

We further compared the frequency of each cell type after BAT injury. On 5 dpi, neutrophils and myeloid-derived cells made up most of the total cell population, while the percentage of FAPs decreased dramatically (Fig. 2D and E). This indicates an immediate response of cell types to BAT damage. From 5 dpi to 14 dpi, the relative fraction of neutrophils and myeloid-derived cells decreased dramatically, while FAPs and T lymphocytes increased (Fig. 2E).

To identify cell populations contributing to brown adipogenesis and BAT regeneration, the expression of adipogenesis-related genes were computed by analyzing data of single-cell RNA-sequencing. Preadipocyte-enriched genes (*Cd38*, *Cd34*, *Ly6a*, and *Pdgfra*), adipogenic master regulators (*Cebpb*, *Cebpa*, and *Pparg*), late adipogenic genes (*Fabp4*), and adipogenic genes (*Fasn*, *Agpat2*, *Lpin1*, and *Scd1*) were widely expressed in FAPs and myeloid-derived cell clusters (Fig. 2F to H and Fig. S3A). Since we have removed muscle from the BAT and isolated pure BAT progenitor cell populations for ScRNA-seq, the level of mature adipocyte markers (*Adipoq*, *Plin1*, and *Lep*) and myogenic transcription factors (*Myod1*, *Myf5*, and *Pax7*) was absent in almost all clusters (Fig. S3A and B). These results suggest that specific cell types, particularly FAPs and myeloid-derived cells expressing lipogenesis-associated genes after BAT injury, may act as progenitors of BAs and participate in BAT regeneration.

### Clustering and pseudotemporal trajectory analysis of FAPs involved in BAT regeneration

**Fig. 3.**
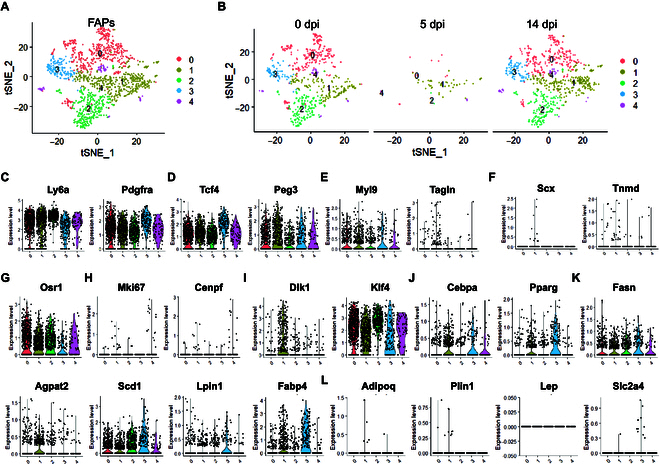
Clustering and pseudotemporal trajectories identify transcriptional dynamics of FAPs. (A and B) Graph clustering of fibroblast/FAPs showing 5 subclusters. (C to L) Expression of different marker genes (*Ly6a*, *Pdgfra*, *Tcf4*, *Peg3*, *Myl9*, *Tagin*, *Scx*, *Tnmd*, *Osr1*, *Mki67*, and *Cenpf*) and adipogenesis-related markers (*Dlk1*, *Klf4*, *Cebpa*, *Pparg*, *Fasn*, *Agpat2*, *Scd1*, *Lpin1*, *Fabp4*, *Adipoq*, *Plin1*, *Lep*, and *Slc2a4*).

Previous studies have demonstrated that new BAs in BAT are derived from resident *Pdgfra*^+^ progenitors under cold stress [20*,*^23^]. To investigate the dynamic characteristics and potential role of FAPs in BAT remodeling, we did subsequent analysis on FAPs. Graph-based clustering identified 5 juxtaposed subclusters (Fig. 3A and B). Visualization of the top 20 most variably expressed genes between subclusters revealed distinct transcriptional profiles of the 5 subgroups (Fig. S4A). All the 5 subclusters expressed high levels of *Ly6a*, *Pdgfra, Osr1*, and *Klf4*, and nearly no expression of mature adipocyte markers (*Adipoq*, *Plin1*, and *Lep*), suggesting that these cells are more primitive undifferentiated cells (Fig. 3C to L). Subcluster 1 was enriched in *Peg3*, *Myl9*, *Tagln*, and activated FAP markers (*Osr1*) (Fig. 3D to G). The top 20 upregulated genes in subcluster 2 showed elevated levels of *Pcsk6* and *Anxa3* (Fig. S4A). Notably, subclusters 1 and 3 had high expression levels of early adipogenic genes (*Dlk1*) and adipogenic genes (*Cebpa* and *Pparg*), respectively (Fig. 3I and J). A fraction of cells in subcluster 3 expressed *Lpin1* and *Fabp4*, but few other fully differentiated markers (Fig. 3K and L). These results indicated that subclusters 1 and 3 were more committed preadipocytes. Cells in subcluster 4 were proliferative and expressed *Mki67* and *Cenpf* (Fig. 3H).

To identify all subpopulations of FAPs that can differentiate into adipocytes, we employed pseudotime trajectory analysis. This enabled us to understand how FAPs progress into adipocyte progenitors and differentiating adipocytes. By ordering cells in pseudotime, we are able to assemble most FAPs into one major trajectory, with 4 bifurcations and 5 stages (Fig. S4B). The proliferative subcluster 2 was located toward the origin of the trajectory, which validated the constructed trajectory. Early (subcluster 0) and later (subcluster 3) adipogenic FAPs were located toward the bifurcation ends of the trajectory. We also computed pseudotime dynamics of markedly affected genes among these 5 subclusters and identified 3 modules based on their pseudotemporal expression profile (Fig. S4C). Gene ontology (GO) analysis showed that genes in modules 2 and 3, which had similar upregulated kinetic trends, were enriched in extracellular matrix/structure organization and cell-substrate adhesion involved in ECM–receptor interaction-related pathways (Fig. S4D to I).

To confirm that FAPs are indeed involved in BAT regeneration, we generated *aP2-Cre^+^ Rosa^iDTR/+^ Pdgfra^GFP/+^* triple heterozygous (*Cre^+^*) and *aP2-Cre^+^/Rosa^iDTR/+^* double heterozygous (*aP2-Cre^+^*) mice to specifically damage BAT with diphtheria toxin (DT) injection (Fig. S5A). The *Rosa^iDTR/+^ Pdgfra^GFP/+^* (*Cre^–^*) and *Rosa^iDTR/+^* (*aP2-Cre*) littermates were recruited as controls. On day 3 (D3) after DT injection, the damaged BAT mass was visibly decreased (Fig. S5B) and the BAT morphology changed dramatically from full of uniformly distributed multicentric adipocytes to a fibrotic appearance with enlarged cavities and massive infiltration of mesenchymal cells (Fig. S5C). *Pdgfra* lineage cells proliferated vigorously in BAT with an increased proportion of GFP^+^/Ki67^+^ cells at D3 after DT injection (Fig. S5D to F). These results suggest that subpopulations of BAs and stromal vascular fraction (SVF) cells are derived from *Pdgfra* lineage precursors.

To further evaluate the contribution of FAPs to BAT regeneration, we conducted lineage tracing studies with the tamoxifen-inducible *Pdgfra^creER^/ROSA^mT/mG^* mice, in which *Pdgfra*-lineage cells were labeled by GFP. Notably, IF of BAT-injured frozen sections showed dynamic changes in the number of *Pdgfra^+^* cells at 0, 5, and 14 dpi (Fig. S6A). The *Pdgfra^+^* cells were obviously reduced after BAT injury and gradually increased during BAT regeneration (Fig. S6A and B). To further determine the proportion of *Pdgfra* lineage that gives rise to BAs, we isolated BAT SVF cells from *Pdgfra^creER^/ROSA^mT/mG^* mice and found about 40% GFP-positive and 60% RFP-negative cells in SVF cells. After adipogenic differentiation, approximately 25.3% of mature adipocytes were derived from *Pdgfra* lineage (Fig. S6C). These results suggest that FAPs contribute to brown adipogenesis and BAT regeneration, but other progenitors can also differentiate into BAs and are involved in this process.

### Myeloid-derived cells are involved in BAT regeneration

**Fig. 4.**
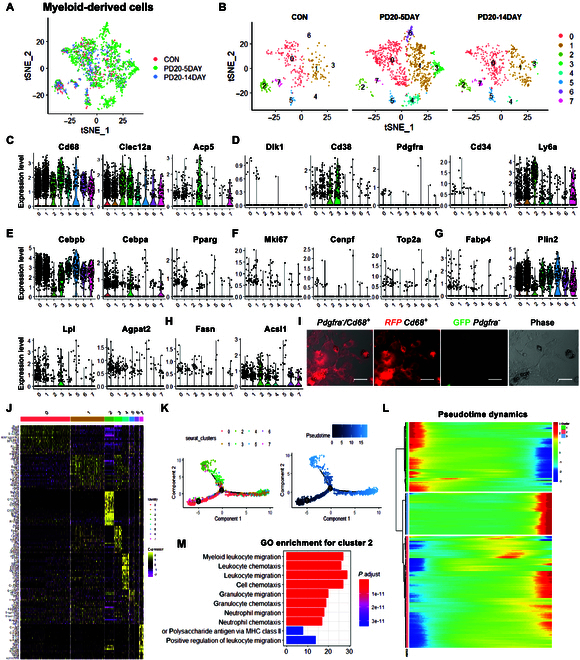
Clustering and pseudotemporal trajectories identified transcriptional dynamics of myeloid-derived cells. (A) The t-SNE plot of merged isolated single cells from normal and BAT-IR-IA-injured BAT. (B) Graph-based clustering of myeloid-derived cells showing 8 subclusters. (C to H) Expression of myeloid-derived cells’ marker genes (*Cd68*, *Clec12a*, and *Acp5*), adipocyte-enriched genes (*Dlk1*, *Cd38*, *Pdgfra*, *Cd34*, and *Ly6a*), adipogenic master regulators (*Cebpb*, *Cebpa*, and *Pparg*), proliferation genes (*Mki67*, *Cenpf*, and *Top2a*), lipid synthesis genes (*Fabp4*, *Plin2*, *Lpl*, and *Agpat2*), and lipid metabolism genes (*Fasn, Acsl1*, *Gpd1*, *Lpin1*, and *Scd1*). (I) Fluorescence and visible light micrographs of all single and *Cd68^+^* cells isolated from BAT-IR-IA-injected BAT of *Pdgfra^cre^/ROSA^mT/mG^* mice after adipogenic differentiation. Red circles: *Pdgfra^−^* cells with lipid droplets; green circles: *Pdgfra^+^* cells with lipid droplets; and yellow circles: *Pdgfra^−^/Cd68^+^* cells with lipid droplets. Scale bars: 50 μm. (J) Heatmap representing the top 10 most differentially expressed genes between macrophage/monocytes sub-clusters identified. Colors and numbers corresponding to the cell clusters shown in J. (K) Pseudotime single-cell trajectory reconstructed by Monocle2 for 8 subclusters of myeloid-derived cells, including Mye0, Mye1, Mye2, Mye3, Mye4, Mye5, Mye6, and Mye7. Pseudotime is colored in a gradient from dark to light blue, and the onset of pseudotime is indicated. Clusters were the modules of genes that co-vary across the pseudotime of myeloid-derived cells. (L) Pseudotemporal heatmap showing gene expression dynamics for marker genes. Genes (rows) were clustered into 3 modules, and cells (columns) were ordered according to pseudotime. The clusters were the cell subclusters of myeloid-derived cells identified by a shared nearest neighbor modularity optimization-based clustering algorithm. (M) GO enrichment analysis of genes in module 2.

To explore other cell populations that might be involved in BAT regeneration, we analyzed the heterogeneous myeloid-derived cells that express adipogenic related genes. We utilized a t-SNE analysis on the cluster and observed 8 juxtaposed subclusters (Fig. 4A and B and S7A). These subclusters were identified based on the profiles of marker genes (Fig. 4C and Fig. S7B to E).

The preadipocyte-enriched genes (*Dlk1*, *Pdgfra*, and *Cd34*) were barely expressed in myeloid-derived cells (Fig. 4D). *Cd38* and *Ly6a* were highly expressed in subclusters Mye2 and Mye3 (Fig. 4D). *Cebpb*, *Cebpa*, and *Pparg*, the adipogenic differentiation regulators, were shown a high expression in Mye0, Mye3, and Mye7 (Fig. 4E). Mye0 and Mye1 cells expressed the proliferation-enriched genes (*Mki67*, *Cenpf*, and *Top2a*) (Fig. 4F). Moreover, *Fabp4*, *Plin2*, *Lpl*, and *Agpat2* were primarily expressed in Mye0, Mye1, Mye3, and Mye5 (Fig. 4G). *Fasn*, *Acsl*, *Gpd1*, *Lpin1*, and *Scd1* were expressed in Mye0, Mye1, Mye2, and Mye7 (Fig. 4H and Fig. S7F). The analysis supported the idea that Mye1 are cycling cells in the G2/M stage (Fig. S7G). A more comprehensive analysis showed the most differentially expressed genes (DEGs) among the 8 subclusters (Fig. 4J).

To explore the relationship between different myeloid-derived subpopulations, we utilized a trajectory inference model to map all the subclusters. Monocle arranged the myeloid-derived cells along a typical trajectory that diverged into 2 distinct branches, with all cells being able to be divided into 3 bifurcations and 8 stages (Fig. 4K). Notably, we observed that Mye0 cells were plotted tightly at the beginning of pseudotime, indicating that these cells possess a more stem-like nature (Fig. S7H).

To better understand the organization of cells in pseudotime, we reanalyzed gene expression profile from non-injured and BAT-IR-IA-injured BAT. We created a heatmap of DEGs displaying the top 10 DEGs and their scaled expression between different states (Fig. 4J). The importantly affected genes among the 8 subclusters along the pseudotime trajectory were assigned to 3 gene modules (Fig. 4L). GO analysis of increased genes in module 2 and module 3 showed a positive regulation of myeloid leucocyte migration and lymphocyte differentiation (Fig. 4M and Fig. S7K). The downregulated genes in module 1 indicated the activation of an immune response (Fig. S7J).

To determine the adipogenic differentiation potential of myeloid-derived cells, we collected *Pdgfra^+^*, *Pdgfra^−^/Cd68^+^*, and *Pdgfra^−^/Cd68^-^* cells from BAT-IR-IA-injured BAT on 5 dpi through magnetic bead cell sorting (MACS). BODIPY staining revealed that a population of *Pdgfra^+^* and *Pdgfra^−^/Cd68^-^* cells could differentiate into mature adipocytes with multiple lipid droplets (Fig. S7I). Notably, several *Pdgfra^−^/Cd68^+^* cells also exhibited small lipid droplets after induction of adipogenesis (Fig. S7I). To further confirm the adipogenic differentiation potential of myeloid-derived cells, we isolated BAT SVF cells from *Pdgfra-mT/mG* mice and sorted out *Pdgfra^−^/Cd68^+^* cells. Consistently, we found that a population of *Pdgfra^−^/Cd68^+^* cells could fully differentiate into matured adipocytes (Fig. 4I). Our in vivo and in vitro data indicate that a subpopulation of myeloid-derived cells have the adipogenic-differentiation potential and could serve as adipogenic progenitors to help BAT regeneration.

### Transcriptome analysis identifies potential regulators in BAT regeneration

**Fig. 5.**
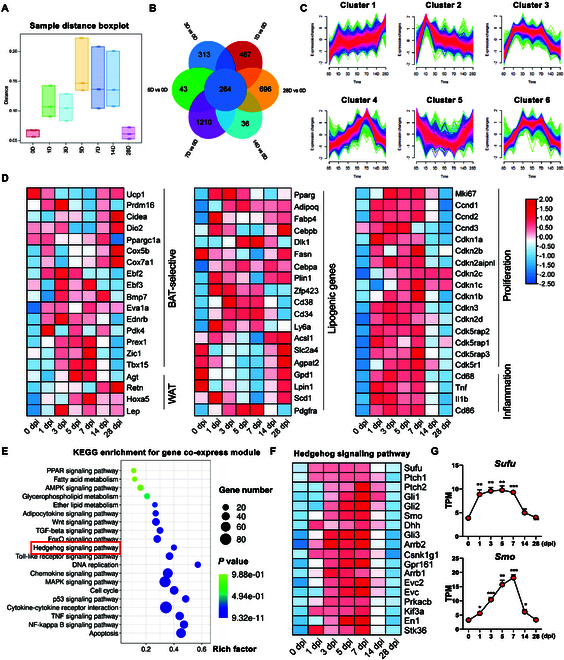
Hedgehog (Hh) signaling is involved in BAT regeneration. (A) Four distances were calculated for multiple groups of samples with different classifications. Inter-sample distance boxplots were drawn to compare the differences in the distribution of distances within and between groups for different samples. (B) Venn diagram of transcriptomic datasets for BAT injury at 0, 1, 3, 5, 7, 14, and 28 dpi. (C) Differential gene module expression trends (6 modules) are depicted as line charts at the indicated time points during BAT regeneration, compared to controls (non-injured, 0 dpi). (D) Heatmap showing relative expression of BAT- and WAT-selective genes, lipogenic, proliferation, and inflammation-related genes from the RNA-seq dataset of BAT-IR-IA-injured vs. non-injured groups. Only genes with *P* < 0.05 are displayed. (E) KEGG enrichment for gene co-express module. (F and G) Heatmap of relative expression of the Hh signaling pathway-related genes derived from the RNA-seq dataset. TPM levels of *Sufu* and *SMO*. *n* = 3. Error bars represent SEM, *P < 0.05, **P < 0.01, ***P < 0.001, 2-tailed Student’s *t*-test.

To better explore the transcriptomic levels during damaged BAT repair, we performed RNA-seq on BAT-IR-IA-injected BAT at different time points (0, 1, 3, 5, 7, 14, and 28 dpi). The boxplots represent the distances between all samples of different categories (Fig. 5A). We compared the BAT-IR-IA injected and non-injected groups, combined Venn diagrams and line plots to show the dynamic gene expression patterns during BAT regeneration, and identified a total of 264 common DEGs and 6 modules (Fig. 5B and C). Among them, the expression of DEGs associated with adipogenesis, inflammation, and proliferation was consistent with our previous qPCR results (Fig. 5D).

To investigate the potential mechanisms of BAT remodeling, GO analysis was examined to distinguish the role of DEGs in the un-injected, and different BAT-IR-IA-injected groups, respectively. The GO analysis revealed that mitochondrial organization (module 1), cell cycle phase transition (module 2), fatty acid metabolic processes (module 3), extracellular matrix organization (module 4), ncRNA metabolic processes (module 5), and ribosome biogenesis (module 6) were obviously altered (Fig. S8A to F). The KEGG pathway showed that thermogenesis, cytokine–cytokine receptor interaction, MAPK, PPAR signaling, ECM–receptor interaction, chemokine, Wnt, FoxO, and Hh signaling pathways were enriched in 6 clusters/modules after BAT injury (Fig. 5E and Fig. S8G to L), suggesting that these signaling pathways were involved in regulating BAT remodeling and repair.

The Hh signaling pathway is known to play a crucial role in various biological processes, including stem cell renewal, cell lineage determination, cell cycle regulation, migration, and mitogenic signaling [24]. In our study, we investigated the expression pattern of Hh-related genes and observed dynamic changes (Fig. 5F and G), indicating that the Hh signaling pathway might be involved in BAT regeneration. We found that BAT injury indeed leads to upregulation of Hh activators and their target genes (Fig. 5F and G). Interestingly, we also found that the suppressor of fused (*Sufu*), a central endogenous inhibitor of Hh in mammals [25*,*^26^], was activated after BAT injury. Previous studies have shown that Hh activation inhibits adipocyte differentiation and tissue regeneration [27*–*31]. We speculated that the upregulation of *Sufu* attenuated Hh activation-inducing impaired BAT regeneration.

### *Myf5*-Cre-mediated deletion of Sufu suppresses BAT development

**Fig. 6.**
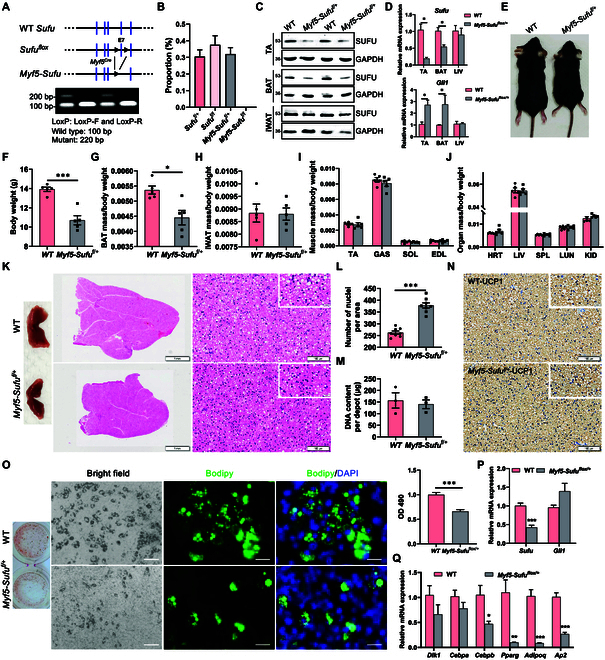
*Myf5-Cre*-mediated deletion of *Sufu* inhibits BAT development. (A) *Myf5*-specific *Sufu* knockout mice were generated by crossing conditional *Sufu^flox/flox^* mice with *Myf5-Cre* transgenic mice. Exon 7 of the mouse Sufu gene was flanked by 2 *LoxP* sites. Vertical lines represent exons and triangles represent *LoxP*. Genotyping analysis with PCR was shown. (B) The genotype distribution ratio of purebred and hybrid genotype mice born offspring, including *Sufu^f/+^*, *Sufu^f/f^*, *Myf5-Sufu^f/+^*, and *Myf5-Sufu^f/f^* mice. (C) Efficient reduction of *Sufu* protein level in TA, BAT, and iWAT of the *Myf5-Sufu^f/+^* mice. (D) The mRNA levels of *Sufu* and *Gli1* in TA, BAT, and liver. *n* = 4. (E) Representative image of WT and *Myf5-Sufu^f/+^* mice. (F to J) Body weight (F, *n* = 5), BAT mass (G), WAT mass (H), muscle mass (I), and other organs’ weight (J), in WT and *Myf5-Sufu^f/+^* mice (4 weeks old). *n* = 5. (K) Representative BAT tissues and H&E staining of BAT sections from WT and *Myf5-Sufu^f/+^* mice at 4 weeks. Scale bars: 1 mm and 100 μm, respectively. (L) The number of nuclei per BAT picture based on (K). Random images from each mouse were chosen. *n* = 8. (M) Genomic DNA content per BAT depot at 4 weeks. *n* = 3. (N) Representative UCP1 immunohistochemistry staining of BAT sections from WT and *Myf5-Sufu^f/+^* mice. (O) Oil Red O and BODIPY staining of total lipids in differentiated BAT SVF obtained from WT and *Myf5-Sufu^f/+^* mice. OD 490 was also measured. (P and Q) mRNA levels of *Sufu*, *Gli1*, and adipogenic related genes (*Dlk1*, *Cebpa*, *Cebpb*, *Pparg*, *Adipoq*, and *Ap2*) after differentiation. *n* = 4. Error bars represent SEM, *P < 0.05, **P < 0.01, ***P < 0.001, 2-tailed Student’s *t*-test. TA, tibialis anterior muscle; Sol, soleus muscle; EDL, extensor digitorum longus muscle; Gas, gastrocnemius muscle; Hrt, heart; Kid, kidney; Liv, liver; Spl, spleen; Lun, lung.

To test above hypothesis, we generated cKO of *Sufu* (Fig. 6A) using *Myf5-Cre* because BAT arises from the *Myf5* lineage precursors **[**10*,*^11^*,*^32^]. We found that the homozygous *of Sufu* cKO (*Myf5-Sufu^flox/flox^*) mice were embryonic lethal (Fig. 6B). Therefore, we used the heterozygous (*Myf5-Sufu^flox/+^*) and the control WT (*Sufu^flox/flox^*) mice for the subsequent study. Western blot and qPCR analysis showed that the *Myf5-Sufu^flox/+^* mice exhibited a targeted deletion of *Sufu* in both BAT and TA muscle, but not in the other tissue, including iWAT and liver (Fig. 6C and D). Notably, qPCR also showed that the *Myf5-Sufu^flox/+^* mice had elevated *Gli1* expression in both muscle and BAT, validating the intended pathway activation in the tissues (Fig. 6D).

Strikingly, the study discovered that *Myf5-Sufu^flox/+^* mice had a smaller size and lower BAT mass than their WT littermates at 4 weeks of age (Fig. 6E to G). However, *Sufu* deficiency did not affect the masses of any other tissues (Fig. 6H to J). The results suggest that *Myf5*-*Cre*-mediated deletion of *Sufu* had a negative impact on BAT development, resulting in a decrease in adipocyte size and higher nuclear densities in the *Myf5-Sufu^flox/+^* mice (Fig. 6K and L). Additionally, the study found that the genomic DNA content per BAT depot was similar between the 2 genotypes, indicating that *Myf5*-specific deletion of *Sufu* did not affect BA cell number (Fig. 6M). In addition, the UCP1 immunohistochemistry (IHC) in BAT was normal in the 2 genotypes (Fig. 6N). These results suggest that the *Myf5-Cre*-mediated deletion of *Sufu* decreased the BA cell size and inhibited BAT development.

To explore whether *Sufu* deficiency affects brown adipogenesis in culture, we collected BAT SVF from WT and *Myf5-Sufu^flox/+^* mice and examined adipogenic differentiation. After induction, we found that the *Myf5-Sufu^flox/+^* had less lipid accumulation and lower mRNA levels of the preadipocyte differentiation transcriptional factors (*Cebpb* and *Pparg*) and the terminal differentiation makers (*Adipoq* and *Ap2*) (Fig. 6O to Q), supporting the notion that *Myf5*-*Cre*-mediated deletion of *Sufu* suppresses brown adipocyte adipogenesis.

## Discussion

Understanding the identity of BAT progenitors and adipocytes emerging from them is vital for manipulating the phenotype of brown adipocytes to improve metabolic health. Previous reports have shown that BAT is degraded by certain environmental stimuli, such as aging and diet-related obesity [8*,*^9^]. Typical BAs are rarely detected in obese individuals because most of them undergo a not yet fully elucidated transition to a “white-like” phenotype that induces inflammatory infiltration of whitening BAT [9]. Therefore, it is important to understand whether and how BAT regeneration is stimulated in adults and the cellular and molecular mechanisms that activate BAT regeneration.

**Fig. 7.**
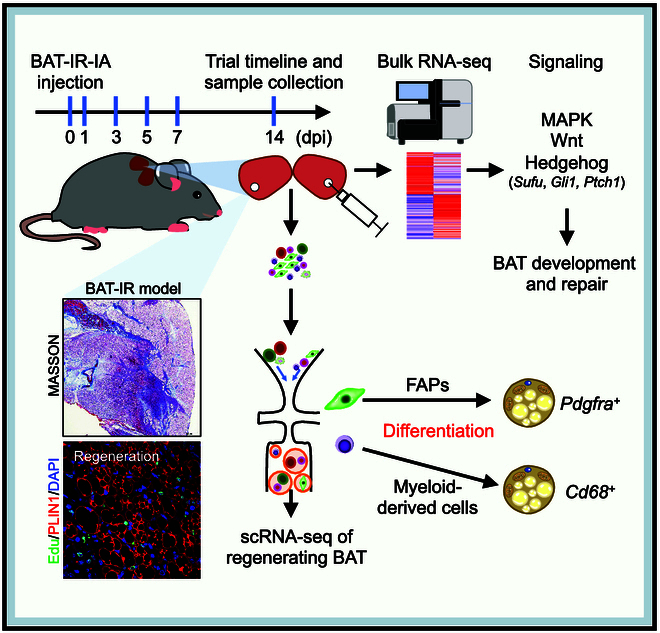
Schema diagram of the cellular and transcriptional dynamics during brown adipose tissue regeneration.

Here, we first established a BAT-IR-IA-induced BAT injury mouse model and found that BAT was efficiently regenerated within 14 days after denaturation. A major drawback of some previous BAT ablation models is that they collectively eliminate progenitor cells and brown fat niches and do not clearly reflect the cellular dynamics of BAT development [21*,*^22^]. In contrast, our BAT injury model preserves both BA progenitor cells and newly regenerated cells and successfully illustrates the dynamics of BAT repair. We also used scRNA-seq to map the cellular composition of BAT, characterize the contribution of each cell type, and determine the cellular origin and dynamics of the progenitors recruited during BAT regeneration. scRNA-seq results and genetic lineage tracing experiment demonstrated the contribution of *Pdgfra^+^* FAPs and *Pdgfra^−^/Cd68^+^* myeloid-derived cells for the emergence of adipocytes in BAT. Furthermore, RNA-seq revealed that signaling pathways related to ECM, cell cycle, and lipid metabolism become greatly altered during BAT remodeling. Additionally, we identified *Hh* pathway as a potential regulator of BAT regeneration and confirmed the regulatory role of *Sufu* in brown adipogenesis using *Myf5-Cre*-mediated deletion of *Sufu*. We revealed previously unknown cellular dynamics and transcriptional regulation program during BAT regeneration (Fig. 7), thus giving rise to a new study model or strategy for studying adult BAT maintenance and function.

In recent years, tissue regeneration has attracted increasing attention [33]. ScRNA-seq has largely contributed to our present understanding of tissue function and homeostasis [1*,*^34^]. Some studies have shown the functional heterogeneity of brown, white, and beige fat [2*,*^19^*,*^35^*,*^36^]. Song et al. [19] previously found that low and high-thermogenic BA subpopulations coexist in mouse BAT. Gallerand et al. [37] uncovered the diversity of immune cells in BAT and emphasized the function of monocyte recruitment in tissue remodeling. However, cellular heterogeneity and dynamic changes in remodeling have not been studied in an adult BAT injury model yet as far as we know. Here, we filled this gap and characterized the cellular diversity of BAT regeneration using 10 major cell types, including FAPs, myeloid-derived cells, neutrophils, T lymphocytes, B lymphocytes, NK cells, myofibroblasts, APCs, fibroblasts, and ECs (Fig. 2C). Among them, FAPs and immune cells exhibited dynamic changes during BAT remodeling.

*Pdgfra^+^* cells have long been the focus of studying muscle-resident precursors of adipocytes and fibroblasts that accumulate in injured skeletal muscle [38*,*^39^]. These cells, called FAPs, can be identified from the expression of *Pdgfra* with *Sca1* and *CD34* and are activated to proliferate in response to tissue damage [38*,*^39^]. Previous studies have identified a role for *Pdgfra* as a cell surface marker of adipogenic precursors in adult WAT and muscle, which led us to investigate its expression during BAT development [23*,*^40^]. Our scRNA-seq data suggest that FAPs are drastically reduced at 5 days after BAT injury and increased continuously between 5 and 14 dpi, suggesting that this cellular subtype may contribute to BAT adipogenesis. Based on clustering and pseudotemporal analysis of FAPs, we revealed the heterogeneity and different cell fate potentials of FAPs during BAT regeneration. Previous studies suggested that *Pdgfra* favors brown adipose precursors in a heterogeneous population of embryonic *Myf5^Cre^* cells [41]. Besides, *Pdgfra*-expressing adipocyte precursor cells (*Pdgfra^+^*/*Sca-1^+^*) can differentiate into beige or brown adipocytes under cold stress [20]. Our in vivo and in vitro experiments revealed that a proportion of *Pdgfra^+^* cells were able to differentiate into mature BAs, suggesting the contribution of FAPs to BAT regeneration.

We also found that a subset of myeloid-derived cells in the brown adipose niche expressed preadipocyte-enriched genes and adipocyte differentiation-related genes. Using MACS and lineage tracing experiments, we performed adipogenic differentiation of *Pdgfra^−^*/*Cd68^+^* cells and demonstrated that a subpopulation of myeloid-derived cells can undergo brown adipogenesis and be involved in BAT repair.

Through transcriptome sequencing, we showed that several signaling pathways including the Hh signaling pathway were dramatically changed. Hh signaling is a conserved evolutionary pathway for controlling tissue development and homeostasis. This is primarily mediated by Gli family transcription factors upon Hh activation [30]. Binding the ligand to the receptor Patched 1 (Ptch1) releases the transmembrane protein Smoothened (Smo) from Ptch1 inhibition. Consequently, Smo interacts with the Gli family of transcription factors (*Gli1*, *Gli2*, and *Gli3*), signaling intracellularly [42]. *Sufu* inhibits Hh signaling through its regulation of *Gli2* and *Gli3*.

Here, we found that BAT injury did cause upregulation of Hh activators and their downstream target genes. Interestingly, we also found that *Sufu*, an endogenous inhibitor of Hh, was activated after acute BAT injury. Previous studies have demonstrated that activation of the Hh pathway inhibits adipocyte differentiation [27*,*^31^*,*^42^]. Additionally, limited Hh signaling in the primary cilia of FAPs facilitates adipogenesis in damaged muscles [28]. Hh activation inhibits the formation of mammalian white adipose tissue (WAT) formation by dysregulating early adipogenic factors including *Pparg*, *Cebpb*, and *Cebpa* [29]. We anticipated that the upregulated expression of *Sufu* might attenuate Hh activation-inducing impaired BAT regeneration. To test our hypothesis, we detected the expression of direct Hh target gene using *Myf5*-*Sufu*-specific knockout (KO) mouse model. We found an elevated *Gli1* expression in BAT of KO mice, validating the intended pathway activation. Furthermore, the *Sufu* KO mice exhibited an inhibited BAT development.

Although the role of the Hh signaling pathway in BAT development has been studied by 2 groups, their results are controversial [29*,*^43^]. Pospisilik et al. [29] previously found the *Ap2-Cre*-mediated *Sufu* deletion model (*Ap2*-*Sufu*) and found that *Sufu* deficiency regulates WAT but not BAT development in cKO mice. The *Ap2*-*Sufu* KO mice exhibited a distinctly lean phenotype with an overall marked reduction in WAT (including subcutaneous, perirenal, and mesenteric fat) mass, but fully developed BAT with normal size and lipid content. They also found that activation of Hh signaling in vivo and in vitro using the Hh activator Smo Agonist prevented white but not brown adipocyte differentiation [29]. Contradictory to their findings, Nosavanh et al. [43] found that brown preadipocytes contain primary cilia and are Hh-responsive, and BAT development is disrupted in *Ap2-Cre*/*Ptch1^flox/−^* and *Ap2-Cre*/*SmoM2* mice. Moreover, they showed that the Hh pathway inhibits brown preadipocyte differentiation, partially via upregulation of chicken ovalbumin upstream promoter transcription factor II [43]. The controversy between the 2 observations may reside in the effect of Hh pathway activation in different mouse models. The phenotype observed in *Ap2*-Sufu KO mice may not reflect the effects of the Hh pathway at maximal levels of activation. In our study, we observed that the cKO (*Myf5-Cre/Sufu^flox/flox^*) mice were embryonic lethal and the *Myf5Cre/Sufu^flox/+^*mice had reduced body weight and BAT mass. Our in vivo and in vitro experiments further demonstrated that *Sufu* depletion suppresses the early stage of brown adipogenesis. Our findings contrast with the phenotype of *Ap2-Cre*-driven Sufu KO mice [29]. We hypothesize that their observations are inconsistent because *Ap2-Cre* primarily mediates the knockdown or knockout of mature adipocytes [44*,*^45^]. BAT is primarily derived from an *Myf5*-positive lineage [11*,*^32^*,*^46^], which mediated the deletion of genes in BAT and skeletal muscle progenitors during embryonic development. These findings suggest that the *Cre* sources may lead to different BAT phenotypes after *Sufu* elimination.

In summary, we provide a comprehensive resource characterizing cellular dynamics and a transcriptomic map of BAT repair. Our map also defines the progenitor cells responsible for BAT development and maintenance. Importantly, we identified the signaling pathway and examined the role of Hh in regulating early BAT development. Our findings provide a new avenue for understanding the molecular features and regulatory mechanisms of BAT development.

## Materials and Methods

### Animals

The study involving mice received approval from the Zhejiang and Purdue University Animal Care and Use Committee. For lineage tracing, mice with specific genetic modifications were used, including *Pdgfra-Cre^ER^* (Stock No. 018280), *ROSA^mT/mG^* (Stock No. 007676), *aP2-Cre^+^* (stock005069), and *Rosa^LSLiDTR/+^* (Stock No. 007900, abbreviated as *Rosa^iDTR/+^*) mice purchased from the Jackson Laboratory (www.jax.org). *Pdgfrα^cre-ER^/ROSA^mTmG^* mice were generated. *Myf5-Sufu^flox/flox^* and *Myf5-Sufu^flox/+^* were produced by crossing *Sufu*-floxed mice with *Myf5*-*Cre* mice.

### Construction of a BAT-IR-IA-induced adult BAT injury model

Mice were placed in the sternal recumbency position and anesthetized. The surgical site was aseptically prepared by shaving the hair from the neck to just below the scapula. Absorbable subcuticular sutures were used to close the incision after injury. Along the length of the BAT on each side, 20 μl of BAT-IR-IA (v/v) (containing 5% phosphatidylcholine and 5% sodium deoxycholate) in sterile 0.9% NaCl was injected. Mice were sacrificed and BAT was harvested immediately for scRNA-seq and other experiments at 0, 1, 3, 5, 7, and 14 dpi.

### DT injection-induced injury of BAT

For the DT injection-induced injury of BAT, *aP2-Cre^+^/Rosa^iDTR/+^* mice were utilized due to the presence of DT receptors in their mature adipocytes. A working solution of DT (Calbiochem) was prepared by dissolving 2 μl of the stock solution (1 mg/μl) in 1 ml of saline. DT (2 ng/μl) in 60 μl of saline was injected into the BAT at 2 different sites to ensure even distribution of the drug and minimize leakage. After DT injection for 3 days, BAT was harvested and subjected for further examination.

### Histology and IF staining

Harvested BAT samples were fixed with 4% paraformaldehyde for 24 h at 4 °C. H&E and IHC staining were performed according to the manufacturer's instructions. Antibodies used are shown in Table S1.

For IF staining, BAT sections or cells were incubated for 1 h after deparaffinization and antigen retrieval with blocking buffer in phosphate-buffered saline containing 5% goat serum, 2% bovine serum albumin, 0.2% Triton X-100, and 0.1% sodium azide. Samples were then incubated overnight at 4 °C with primary antibodies to UCP1 (ab10983, 1:200), collagen I, Ki67 (ab15580, 1:200), and PLIN1 (D1D8, 1:200). After washing 3 times, samples were incubated with the secondary antibody for 1 h at room temperature. Samples were then incubated with the fluorescent dye 4'-6-diamidino-2-phenylindole (DAPI) for 10 min to expose nuclei. Fluorescence images were single-channel grayscale images taken with a Leica DM 6000B fluorescence microscope (Leica Microsystems, Wetzlar, Germany) with a ×20 objective (numerical aperture, 0.70).

### Single-cell RNA-seq using 10X genomics chromium

We obtained samples from mice that were treated experimentally and conducted scRNA-seq on all living cells isolated from BAT that was either non-injured (0 dpi) or injured with BAT-IR-IA at 5 dpi and 14 dpi. The BAT samples were enzymatically dissociated using collagenase I (0.15 g per 100 ml) for 30 min and filtered using a 40-μm cell sieve. The BAT SVFs were isolated and processed to remove erythrocytes and dead cells. Each single-cell suspension was assessed for viability, and the corresponding volume was calculated. scRNA was sequenced using 10X Genomics' v3 kit following their protocol. Illumina NovaSeq platform was used for library construction (LC-Bio Technology Co., Ltd., Hangzhou, China). The resulting reads from the Illumina NovaSeq platform were aligned and the number of unique molecular identifiers at the gene level was determined using Cell Ranger. The number of cells captured, number of reads, and other key information of the scRNA-seq data are provided in Table S2.

### RNA-seq analysis

BAT samples were harvested at certain times (0, 1, 3, 5, 7, 14, and 28 dpi) after BAT-IR-IA injection. RNA collection and RNA-seq were conducted at Sangon Biotech (Shanghai, China). The quality of the sequencing data was assessed using FastQC (version 0.11.2). Transcribed gene expression levels were calculated using StringTie software (version 1.3.3b). DESeq2 R (1.16.1) software package was used for differential expression analysis between groups. Genes were considered as differentially expressed if *q* value < 0.05 and |foldchange| > 1.5.

### Statistical analysis

The data were presented as means ± standard error of the mean (SEM) from at least 3 independent experiments. GraphPad Prism 8.0 software was used to prepare graphs and analyze the data. Two-tailed Student's *t*-tests were used for comparisons and differences between groups were considered significant at *P* < 0.05.

## Data Availability

All data are available from the corresponding authors upon request.
