# Supplementary material for: Cellular and Transcriptional Dynamics during Brown Adipose Tissue Regeneration under Acute Injury
Source: Research (Wash D C). 2023 Nov 8;6:0268. doi: 10.34133/research.0268 (PMC10907023; doi:10.34133/research.0268)
Supplement: Supplementary 1 — Figs. S1 to S8 Tables S1 and S2 Supplementary methods [file research.0268.f1.docx]

**Supplementary Materials**

Figs. S1 to S8.

Tables S1 and S2.

Supplementary methods.

**Supplementary Figure 1**

**
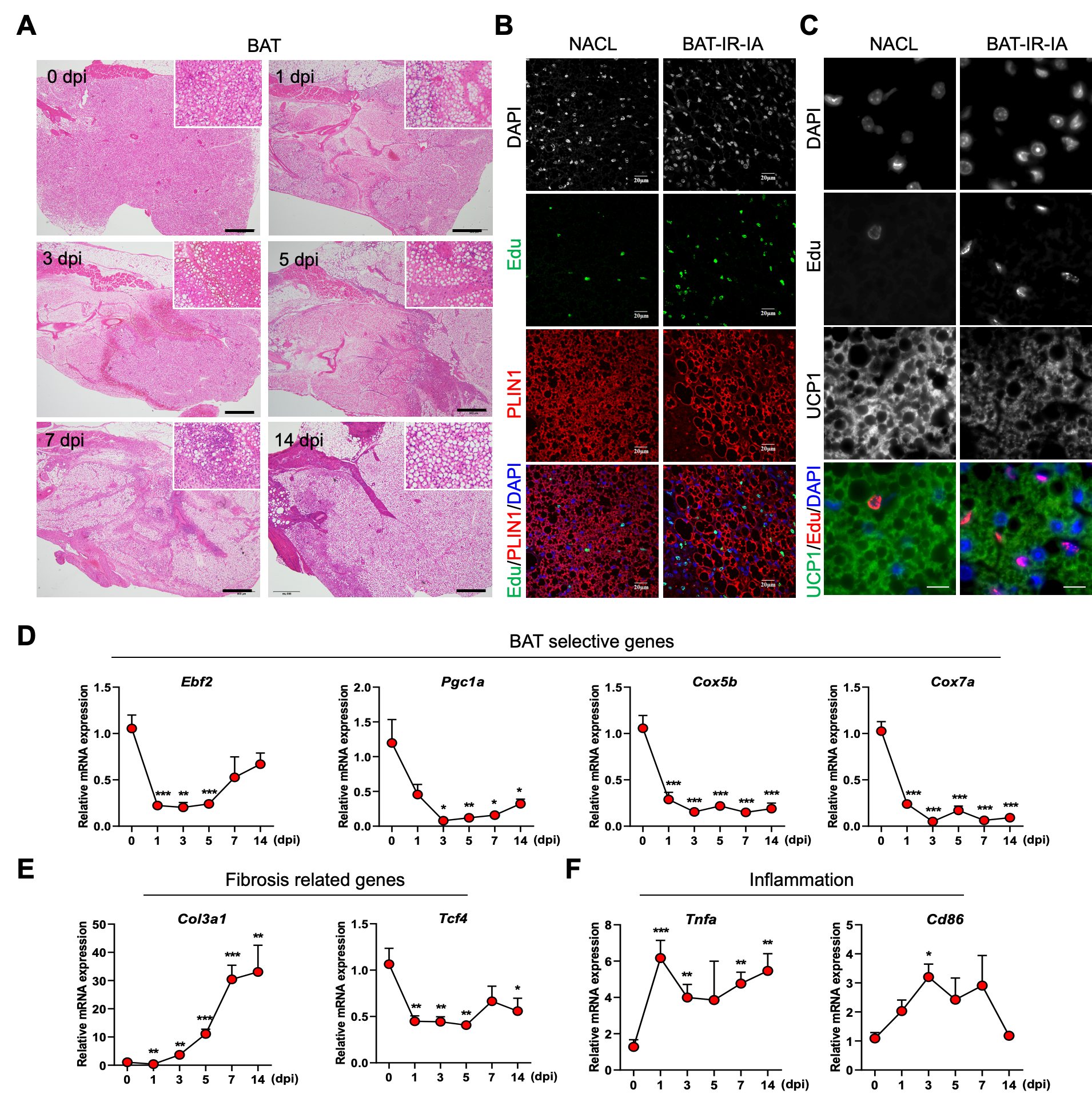
**

**Fig. S1. Morphological changes and gene expression patterns** **in BAT-IR-IA-induced BAT injury model, related to Figure 1.** (A) Cross-sectioning and hematoxylin & eosin staining of BAT at the selected time point to confirm features of BAT regeneration dynamic. Scale bar: 200 μm. (B) Immunofluorescence staining of Perilipin-1 and DAPI with EdU labeling on BAT sections. Scale bar, 20 μm. Immunofluorescence for proliferating cells (EdU, green) and adipocytes (PLIN1, red) in BAT at 14 dpi with BAT-IR-IA injection. Nuclei were stained with DAPI. Scale bars: 20 μm. (C) Immunofluorescence staining of UCP1 (Green) and DAPI with EdU (Red) labeling on BAT sections at 14 dpi with BAT-IR-IA injection. (D-F) Gene expression of BAT-selective (D), fibrosis (E), and inflammation (F), markers, supplement to Figure 1. n=6. Error bars represent SEM, **P*<0.05, ***P*<0.01, ****P*<0.001, two-tailed Student’s t-test.

**Supplementary Figure 2**

**
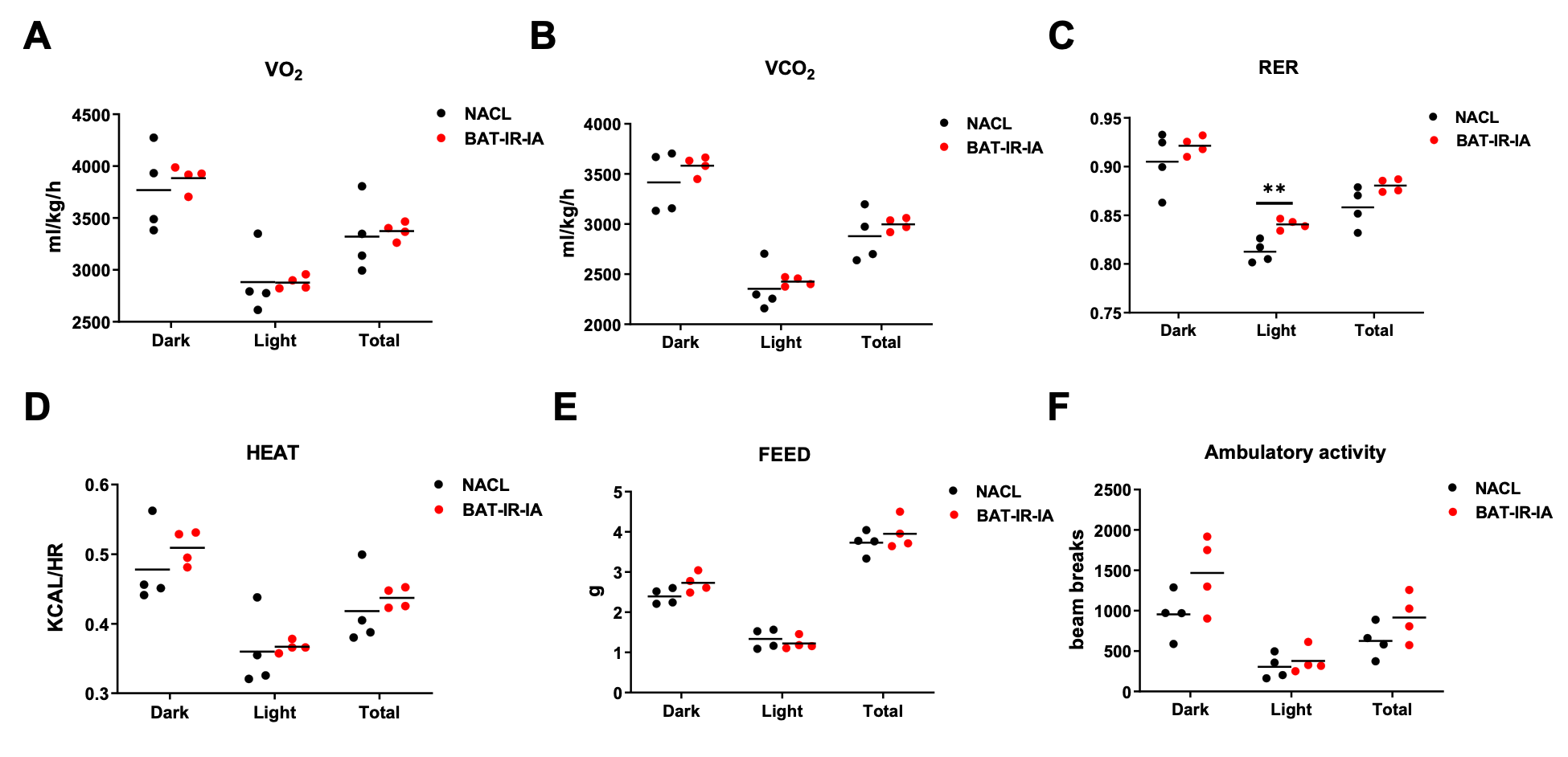
**

**Fig. S2.** **Metabolic effects in BAT-IR-IA-induced BAT injury model.** The indirect calorimetry study was measured using an indirect calorimetry system (Oxymax, Columbus Instruments), installed under a constant environmental temperature (24 ℃) and a 12-h light (06:00-18:00 hours), 12-h dark cycle (18:00-06:00 hours). (A-F) Average day and night-time oxygen consumption (VO_2_) (A), CO_2_ production (VCO_2_) (B), VCO_2_/VO_2_ (RER) (C), heat production (D), food intake (E), and ambulatory activity (F). Error bars represent SEM, **P* < 0.05, ***P* < 0.01, two-tailed Student’s t-test.

**Supplementary Figure 3**

**
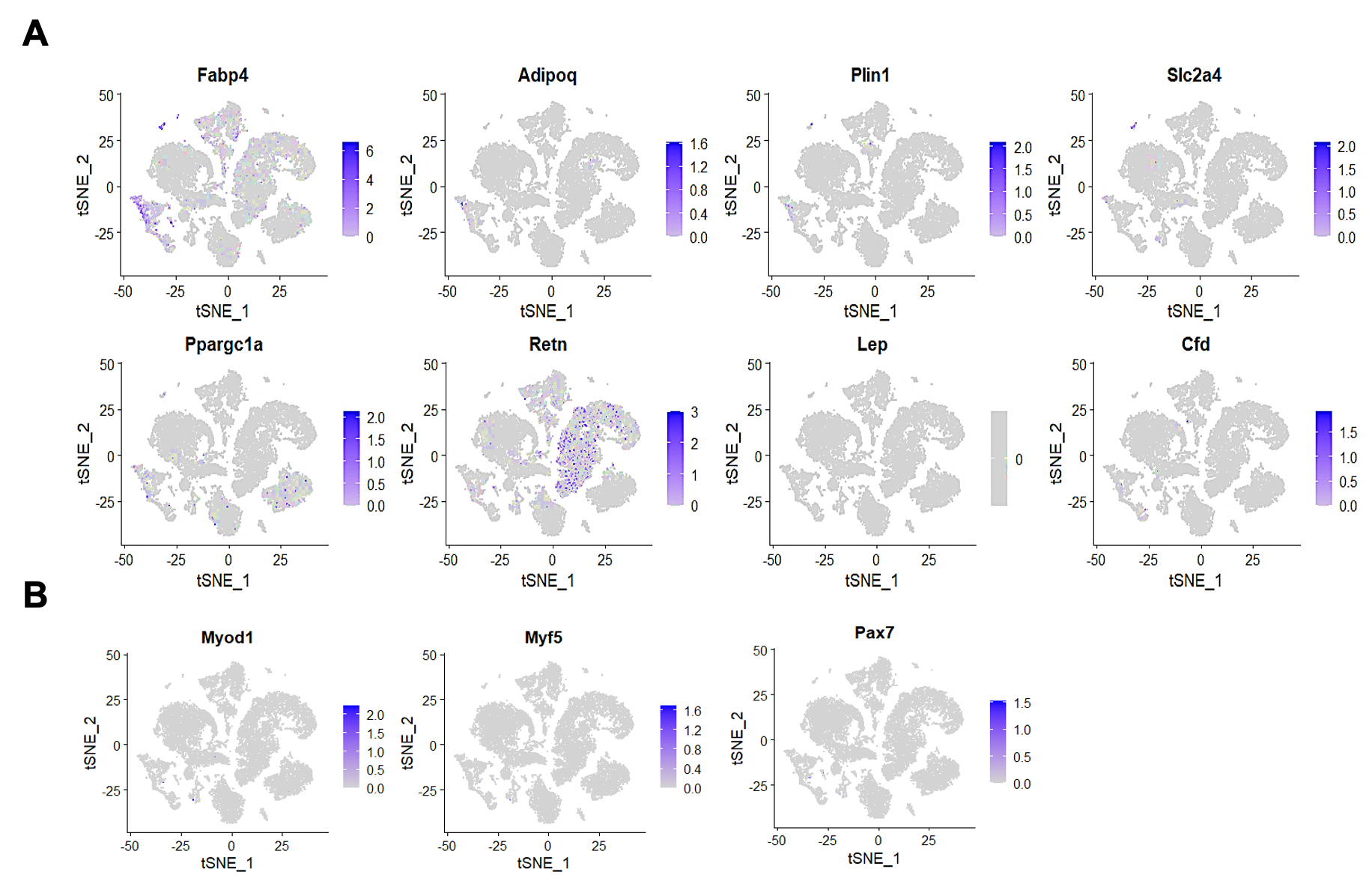
**

**Fig. S3. Gene expression profiles by scRNA-seq during BAT regeneration.** (A) Gene expression distribution of mature adipocyte marker genes (*Fabp4*, *Adipoq*, *Plin1*, *Slc2a4*, *Retn*, *Lep*, and *Cfd*) in these populations. (B) Gene expression distribution of myogenic genes (*Myod1*, *Myf5*, and *Pax7*) in the populations.

**Supplementary Figure 4**

**
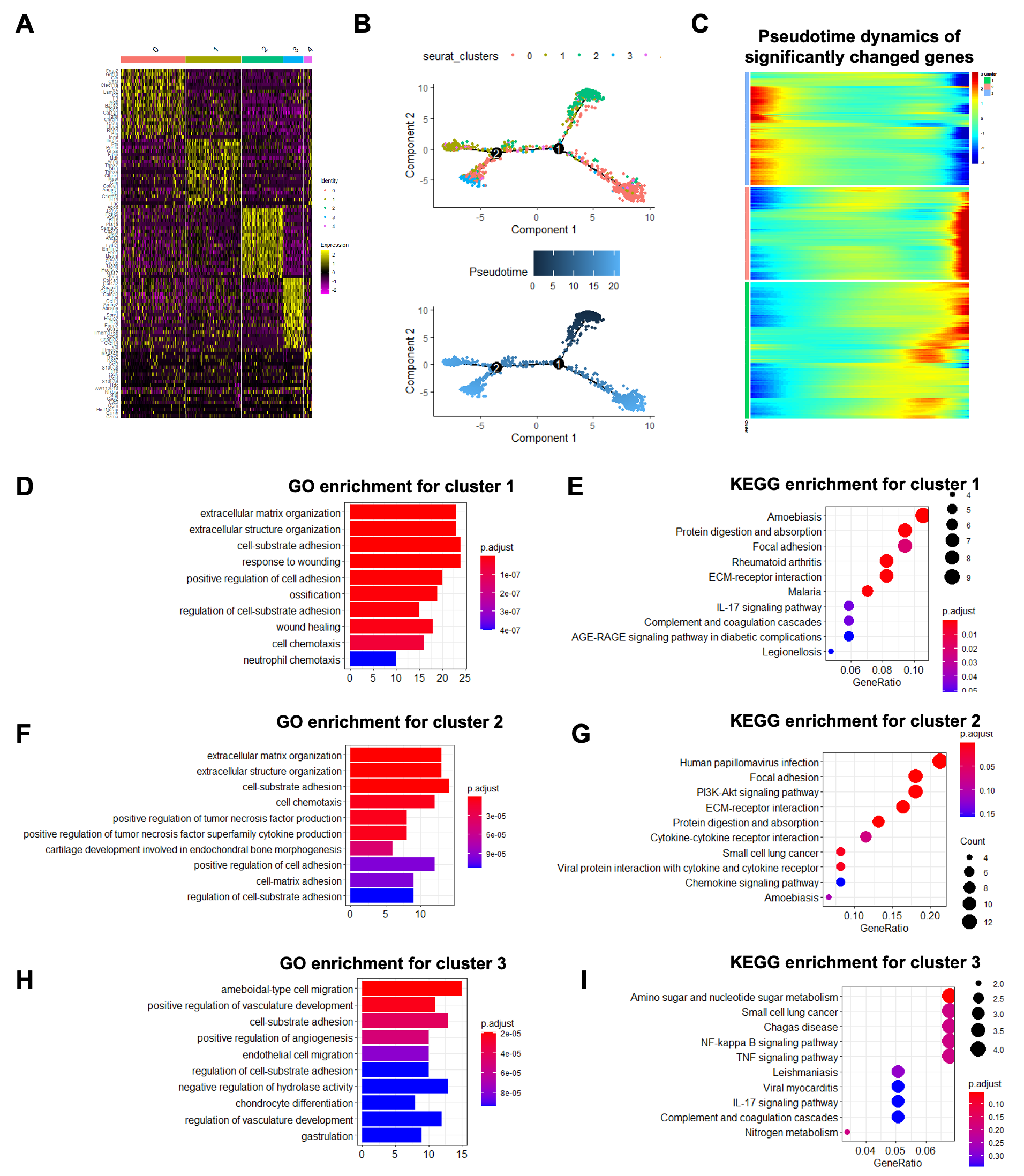
**

**Fig. S4. Clustering and pseudotemporal trajectories identify transcriptional dynamics of FAPs.** (A) Heatmap of top 20 significant genes between intact, BAT-IR-IA-injured (5 dpi), and BAT-IR-IA-injured (14 dpi) FAPs. (B) Pseudotime single-cell trajectory reconstructed by Monocle2 for fibroblast/FAPs. Pseudotime is colored in a gradient from dark to light blue, and the start of pseudotime is indicated. Clusters were the modules of genes that co-vary across the pseudotime of FAPs. (C) Pseudotime single-cell trajectory reconstructed by Monocle2 for FAPs. Pseudotime single-cell trajectories for each subcluster of FAPs. The clusters were the cell subclusters of FAPs identified by a shared nearest neighbor (SNN) modularity optimization based clustering algorithm. (D-I) GO and KEGG enrichment analysis for the three clusters in (C).

**Supplementary Figure 5**

**
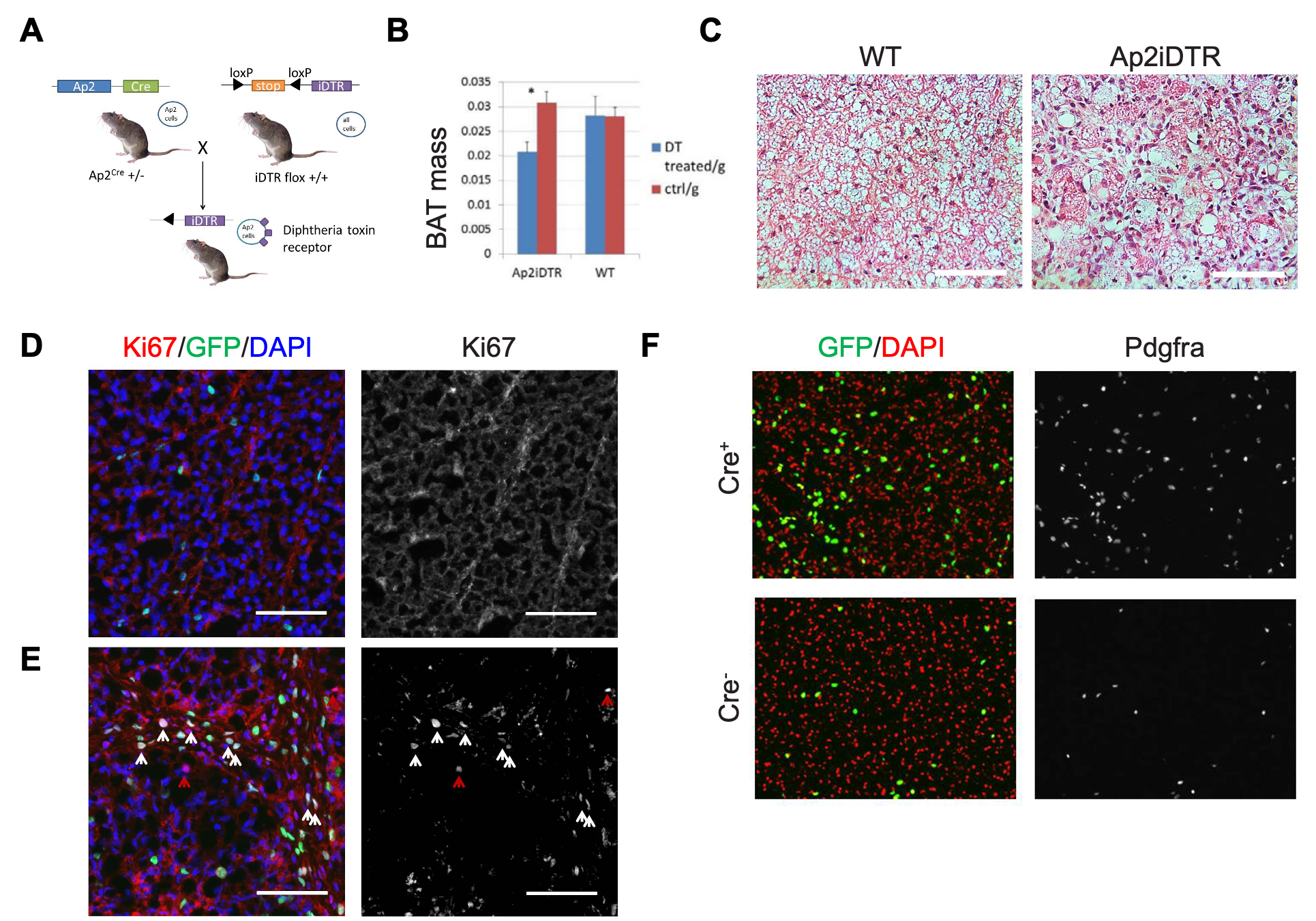
**

**Fig. S5. BAT is effectively damaged by genetic ablation DT-treated BAT on D3 after injection.** (A) The breeding strategy of *aP2-cre^+^/Rosa^iDTR/+^* (*aP2-Cre^+^*) mice. *Rosai^DTR/+^* (*aP2-Cre*) littermates are controls. (B) DT or saline (control) treated BAT weight. n=5. (C) H&E sections of DT treated BAT on D3 after injection. Scale bar: 10 μm. (D and E) Proliferating GFP^+^ cells in BAT from *aP2-Cre^+^ Rosa^iDTR/+^ Pdgfra^GFP/+^* mouse (D), and *Rosa^iDTR/+^ Pdgfra^GFP/+^* mouse (E), after DT treatment. White arrows indicate ki67^+^/GFP^+^ cells, and red arrows indicate Ki67^+^/GFP^-^ cells. Scale bar: 10 μm. (F) Picture of GFP^+^ cells in *aP2-Cre^+^/Rosa^iDTR/+^/Pdgfra^GFP/+^* and *Rosai^DTR/+^/Pdgfra^GFP/+^* mouse BAT. n=3.

**Supplementary Figure 6**

**
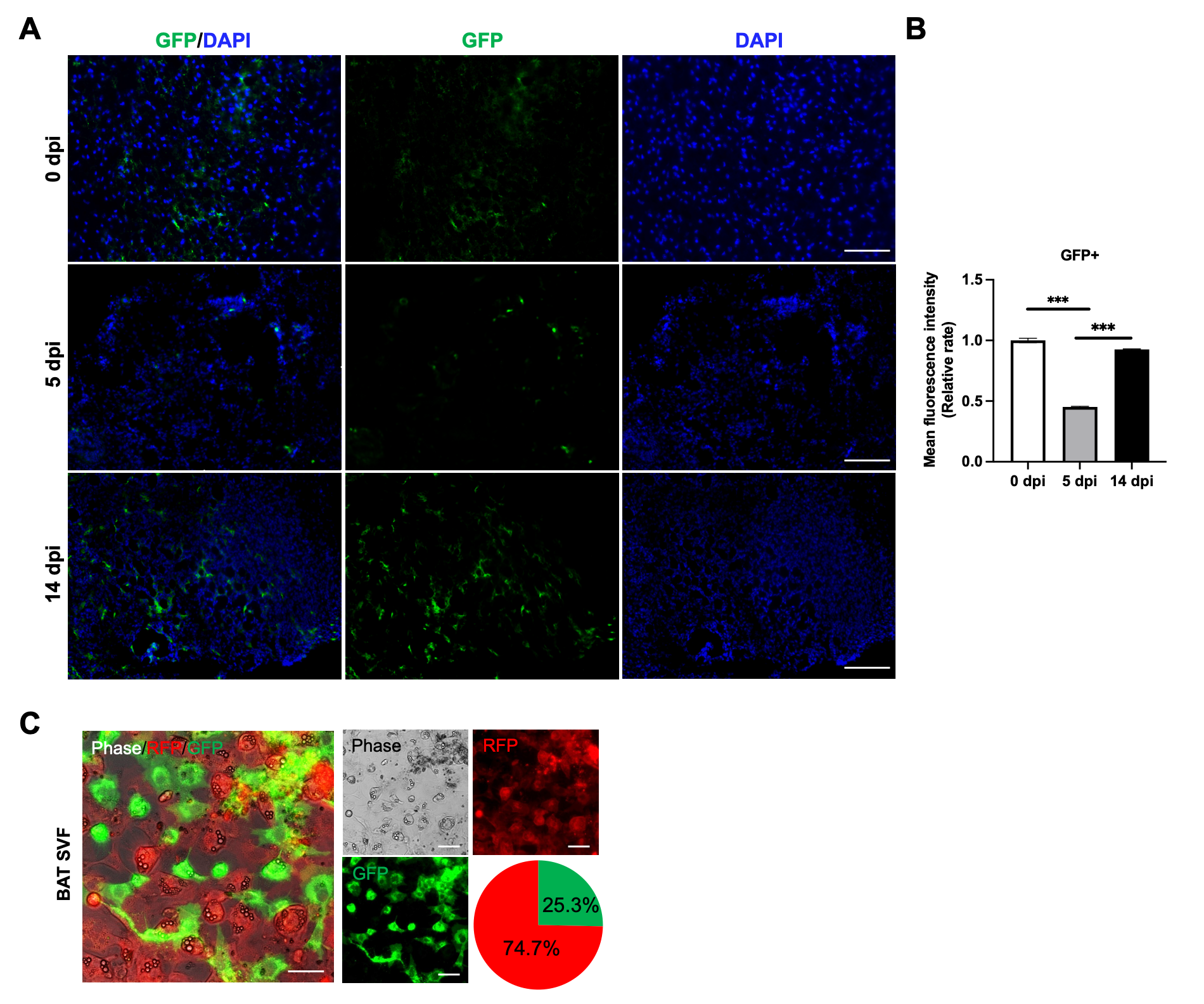
**

**Fig. S6. Detection of Pdgfra lineage origin of BAT.** (A) Results are based on *Pdgfra^cre^/ROSA^mT/mG^* mice at 0, 5, and 14 dpi with BAT injury. *Pdgfra-* lineage cells are labeled in Green (mG, membrane-GFP). Nuclei are stained with DAPI. Scale bar is 50 μm. (B) Quantification of mean fluorescence intensity by ImageJ software based on Figure S6A. (C) The fluorescence and visible light micrographs of BAT SVF isolated from *Pdgfra^cre^/ROSA^mT/mG^* mice after adipogenic differentiation. RFP-positive and RFP-negative adipocyte quantification of the BAT SVF after differentiation. Red circles: *Pdgfra^-^* cells with lipid droplets; green circles: *Pdgfra^+^* cells with lipid droplets. Scale bar: 50 μm.

**Supplementary Figure 7**

**
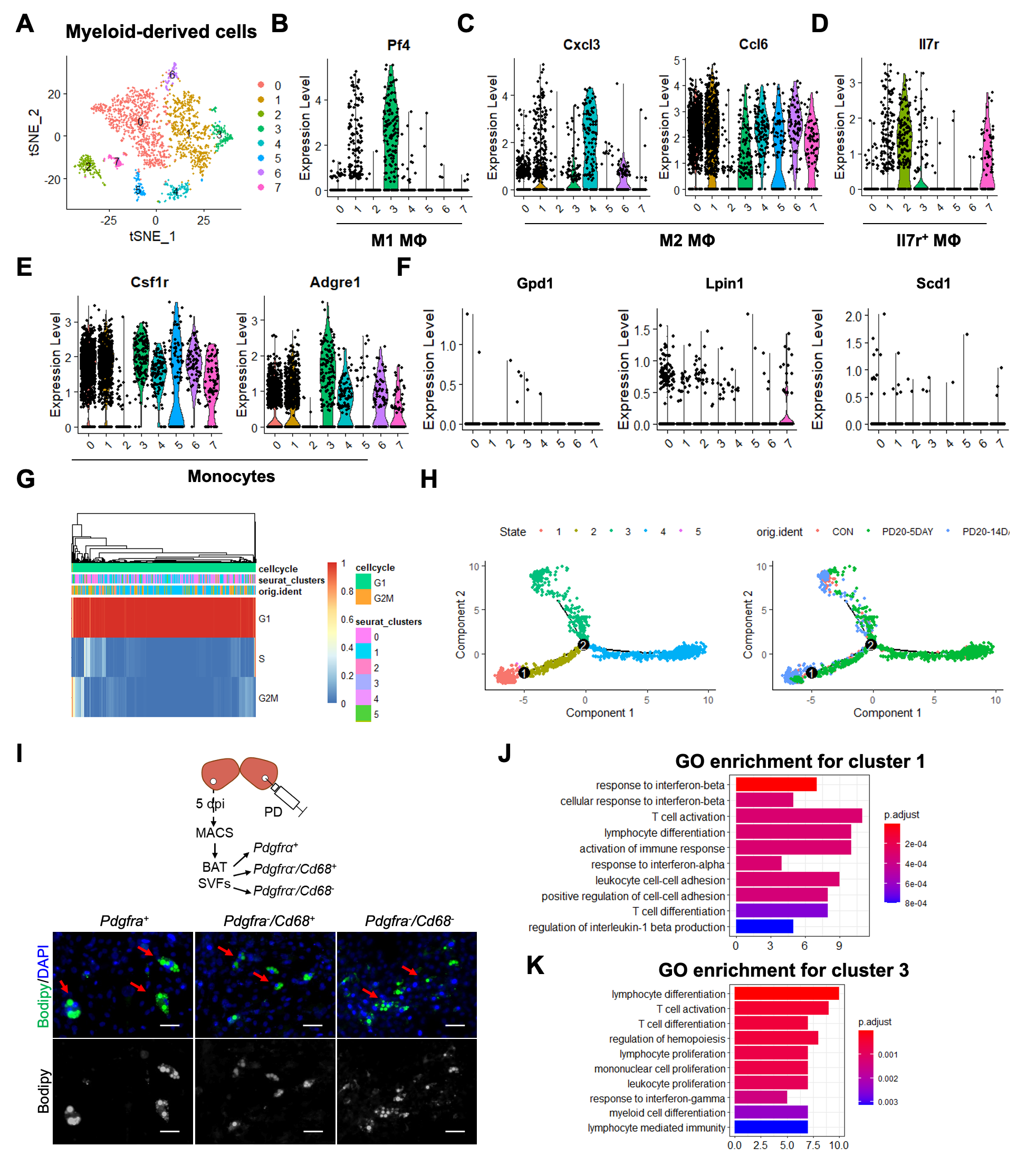
**

**Fig. S7. Clustering and pseudotemporal trajectories identified transcriptional dynamics of** **myeloid‐derived cells.** (A) Graph-based clustering of myeloid-derived cells showing eight subclusters. (B-E) Expression of M1 macrophage (M1 MΦ; *Fabp4*, *Pf4*), M2 MΦ (*Cxcl3*, *Ccl6*), Il7r^+^ MΦ (*Il7r*), monocytes (*Csf1r*, *Adgre1*). (F) Expression of *Gpd1*, *Lpin1*, and *Scd1*. (G) Cell cycle analysis of myeloid-derived cells in BAT. (H) Pseudotime single-cell trajectory is colored by states. (I) Fluorescence light micrographs and phase-contrast images of *Pdgfra^+^*, *Pdgfra^-^/Cd68^-^*, and *Pdgfra^-^/Cd68^-^* cells isolated from BAT-IR-IA-injected BAT of wild-type mice after adipogenic differentiation incubating with Bodipy (green; lipid droplets) and DAPI (blue; nucleus). Scale bar: 50 μm. (J and K) GO analysis for Cluster 1 (J), and cluster 3 (K), were shown respectively.

**Supplementary Figure 8**

**
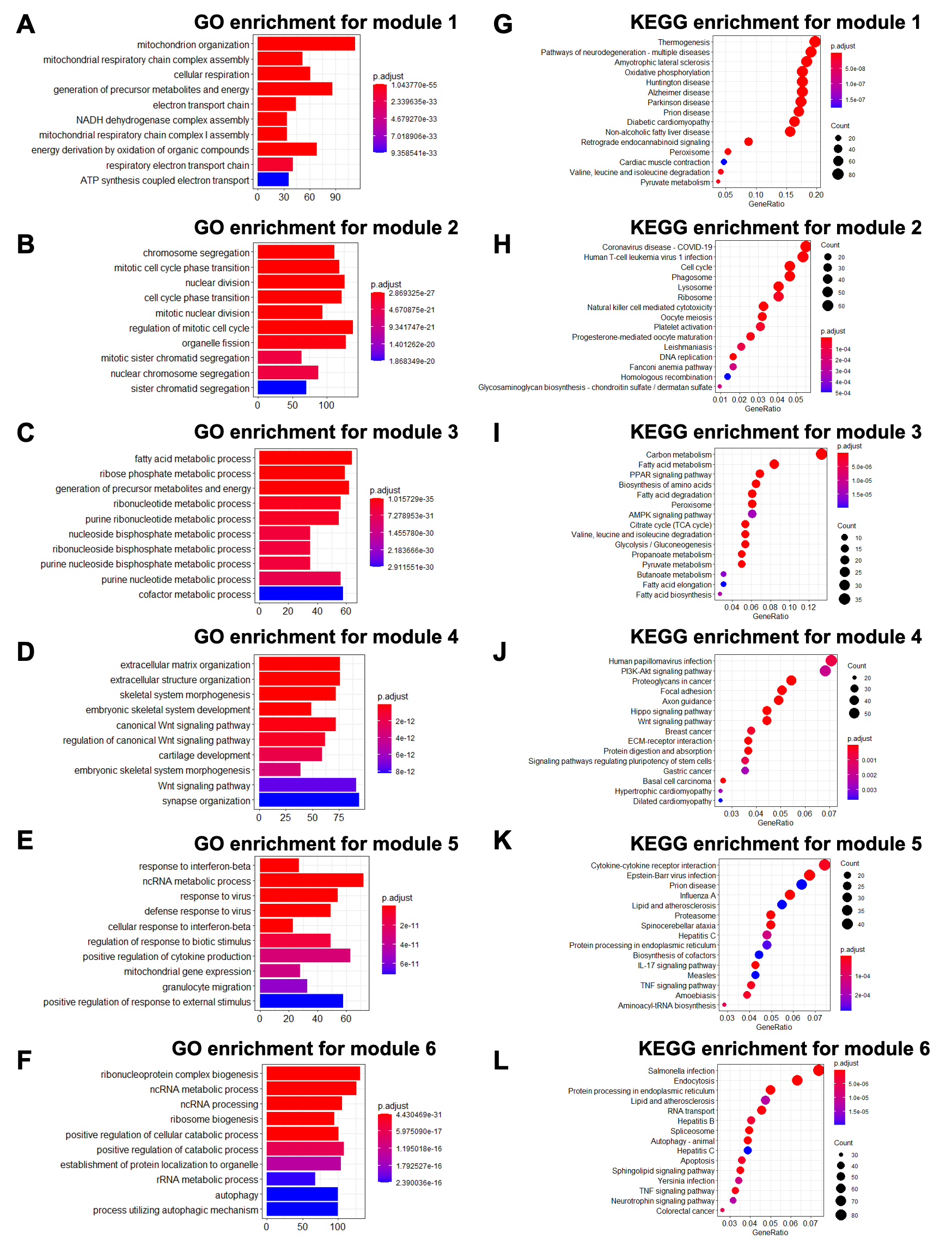
**

**Fig. S8. BAT-IR-IA-induced BAT injury affects transcriptomic profiles.** (A-F) Gene Ontology (GO) enrichment analysis of significantly different genes in NACL- versus BAT-IR-IA-injected BAT for all six modules, based on Figure 5C. The triangle size indicates the significance and corresponding significance values displayed as log10 (*P*-value). (G-L) Functional enrichment analyses were generated using the Kyoto Encyclopedia of Genes and Genomes (KEGG) correspondingly. The triangle size reflects the significance and corresponding values displayed as log10 (*P*-value). The correlation of Top 15 KEGG enrichment pathways.

**Table S1**

| **Reagent or resource** | **Source** | **Identifier** |
| --- | --- | --- |
| **Antibodies** |  |  |
| Anti-Pdgfra (CD140a) | Miltenyi Biotec | Cat#130-101-502 |
| Anti-CD68 | Miltenyi Biotec | Cat#130-101-957 |
| Anti-Collagen I | Servicebio | Cat#GB11022-3-100 GB11022-3 |
| Anti-Ki67 | Abcam | Cat#ab15580 |
| Anti- Myh1e (MF-20) | DSHB |  |
| Anti-PLIN1 | Cell Signaling Technology | Cat#D1D8 |
| Anti-UCP1 | Abcam | Cat#ab10983 |
| Goat Anti-Mouse IgG (H+L)-HRP Conjugate | Invitrogen | Cat#A28177 |
| Goat Anti-Rabbit IgG (H+L)-HRP Conjugate | Invitrogen | Cat#A27036 |
| Goat Anti-Rabbit IgG H&L (Alexa Fluor® 488) | Abcam | Cat#ab150077 |
| Goat Anti-Rabbit IgG H&L (Alexa Fluor® 594) | Abcam | Cat#ab150080 |
| Goat Anti-Mouse IgG H&L (Alexa Fluor® 488) | Abcam | Cat#ab150113 |
| Goat Anti-Mouse IgG H&L (Alexa Fluor® 594) | Abcam | Cat#ab150116 |
| **Cell lines** |  |  |
| Mice BAT SVFs | This study | N/A |
| *Myf5-Sufu^f/+^*, *Sufu^f/f^* BAT SVFs | This study | N/A |
| **Cell culture reagents** |  |  |
| Dulbecco’s Modified Eagle’s Medium-high Glucose (DMEM) | Gibco^TM^ | Cat#11995040 |
| Phosphate buffer solution (PBS) | Servicebio | Cat#G4202 |
| Collagenase I | Gibco^TM^ | Cat#17100017 |
| Fetal bovine serum (FBS) | Gibco^TM^ | Cat#10099141 |
| Super horse serum | Sangon Biotech | Cat#E510006-0100 |
| Penicillin/streptomycin | Sangon Biotech | Cat#E607011-0100 |
| Trypsin EDTA | Sigma-Aldrich | Cat#T4049 |
| Insulin solution human | Sigma-Aldrich | Cat#I9278 |
| Dexamethasone (DEX) | Sangon Biotech | Cat#A601187-0005 |
| 3-isobutyl-methylxanthine (IBMX) | Sangon Biotech | Cat#A606630-0100 |
| Rosiglitazone | Sigma-Aldrich | Cat#R2408 |
| Oil Red O | Sangon Biotech | Cat#A600395-0050 |
| **Chemicals, peptides, and recombinant proteins** | |  |
| BODIPY 493/503 | Thermo Fisher Scientific | Cat#D3922 |
| Bovine Serum Albumin | GEMINI | Cat#700-107P |
| DAPI | Sigma-Aldrich | Cat#D9542 |
| TRIzoL | Invitrogen | Cat#15-596-018 |
| Tamoxifen | Sigma-Aldrich | Cat#T5648 |
| **Critical commercial assays** |  |  |
| First Strand cDNA Synthesis Kit | Thermo Fisher Scientific | Cat#K1631 |
| Dead Cell Removal Kit | Miltenyi Biotec | Cat#130-090-101 |
| MidiMACS™ Separator | Miltenyi Biotec | Cat#130-042-302 |
| MACS® BSA Stock Solution | Miltenyi Biotec | Cat#130-091-376 |
| MS Columns | Miltenyi Biotec | Cat#130-042-201 |
| Red Blood Cell Lysis Solution (10×) | Miltenyi Biotec | Cat#130-094-183 |
| **Western blot reagents** |  |  |
| RIPA buffer | Sigma-Aldrich | Cat#R0278 |
| PVDF membrane | Millipore | Cat#ISEQ00010 |
| Protease inhibitor | Thermo Fisher Scientific | Cat#A32963 |
| Pierce BCA assay kit | Thermo Fisher Scientific | Cat#23225 |
| **Deposited data** |  |  |
| Mice BAT samples RNA-Seq data | This study |  |
| Single cell RNA-seq data | This study |  |
| **Oligonucleotides for qPCR** |  |  |
| Primers for 18s qPCR-F:  TTCTGGCCAACGGTCTAGACAAC | TTCTGGCCAACGGTCTAGACAAC | N/A |
| Primers for 18s qPCR-R:  CCAGTGGTCTTGGTGTGCTGA | CCAGTGGTCTTGGTGTGCTGA | N/A |
| Primers for Adipoq qPCR-F:  TGTTCCTCTTAATCCTGCCCA | TGTTCCTCTTAATCCTGCCCA | N/A |
| Primers for Adipoq qPCR-R:  CCAACCTGCACAAGTTCCCTT | CCAACCTGCACAAGTTCCCTT | N/A |
| Primers for Agt qPCR-F: | TCTCCTTTACCACAACAAGAGCA | N/A |
| Primers for Agt qPCR-R: | CTTCTCATTCACAGGGGAGGT | N/A |
| Primers for Ap2 qPCR-F:  ACCGCAGACGACAGGAA | AAGGTGAAGAGCATCATAACCCT | N/A |
| Primers for Ap2 qPCR-R:  CTCATGCCCTTTCATAAAC | TCACGCCTTTCATAACACATTCC | N/A |
| Primers for Cd68 qPCR-F:  CAAGAACAGCAACGAGTACCG | This study | N/A |
| Primers for Cd68 qPCR-R:  GTCACTGGTCAACTCCAGCAC | This study | N/A |
| Primers for Cd86 qPCR-F: | CACGAGCTTTGACAGGAACA | N/A |
| Primers for Cd86 qPCR-R: | TTAGGTTTCGGGTGACCTTG | N/A |
| Primers for Cebpa qPCR-F:  CAAGAACAGCAACGAGTACCG | CAAGAACAGCAACGAGTACCG | N/A |
| Primers for Cebpa qPCR-R:  GTCACTGGTCAACTCCAGCAC | GTCACTGGTCAACTCCAGCAC | N/A |
| Primers for Cebpb qPCR-F:  CAAGAACAGCAACGAGTACCG | ACCGGGTTTCGGGACTTGA | N/A |
| Primers for Cebpb qPCR-R:  GTCACTGGTCAACTCCAGCAC | CCCGCAGGAACATCTTTAAGTGA | N/A |
| Primers for Col1a1 qPCR-F:  CAAGAACAGCAACGAGTACCG | GCTCCTCTTAGGGGCCACT | N/A |
| Primers for Col1a1 qPCR-R:  GTCACTGGTCAACTCCAGCAC | CCACGTCTCACCATTGGGG | N/A |
| Primers for Col3a1 qPCR-F: | CTGTAACATGGAAACTGGGGAAA | N/A |
| Primers for Col3a1 qPCR-R: | CCATAGCTGAACTGAAAACCACC | N/A |
| Primers for Cox7a qPCR-F: | GCTCTGGTCCGGTCTTTTAGC | N/A |
| Primers for Cox7a qPCR-R: | GTACTGGGAGGTCATTGTCGG | N/A |
| Primers for Cox5b qPCR-F:  CAAGAACAGCAACGAGTACCG | TTCAAGGTTACTTCGCGGAGT | N/A |
| Primers for Cox5b qPCR-R:  GTCACTGGTCAACTCCAGCAC | CGGGACTAGATTAGGGTCTTCC | N/A |
| Primers for Ebf2 qPCR-F: | AAGCAACCTCCTTCAAACTTGAG | N/A |
| Primers for Ebf2 qPCR-R: | CATTCTCCACAAAGTCCACGAA | N/A |
| Primers for Ehmt1 qPCR-F: | AAGAGACCAAGCAGGATTGC | N/A |
| Primers for Ehmt1 qPCR-R: | TGTGGAACCTTCATCAGCAG | N/A |
| Primers for Gli1 qPCR-F: | CAAGGCCTTTAGCAATGCCAGTGA | N/A |
| Primers for Gli1 qPCR-R: | ATGCACTGTCTTCACGTGTTTGCG | N/A |
| Primers for Gli2 qPCR-F: | GGTGTGGACTCATTGCCTGA | N/A |
| Primers for Gli2 qPCR-R: | ATGCACCAAATTTACTGCCTGG | N/A |
| Primers for Il6 qPCR-F:  ACGTGGTACTGTGCCTGCTG | CTGGTGACAACCACGGCCTCCCCT | N/A |
| Primers for Il6 qPCR-R:  CGCAGGATGTTGATGTCGTT | ATGCTTAGGCATAACGCACTAGGT | N/A |
| Primers for Leptin qPCR-F:  GAGACCCCTGTGTCGGTTC | GAGACCCCTGTGTCGGTTC | N/A |
| Primers for Leptin qPCR-R:  CTGCGTGTGTGAAATGTCATTG | CTGCGTGTGTGAAATGTCATTG | N/A |
| Primers for Mki67 qPCR-F: | CTGGTCACCATCAAGCGGAG | N/A |
| Primers for Mki67 qPCR-R: | CAATACTCCTTCCAAACAGGCAG | N/A |
| Primers for Myf5 qPCR-F: | AAACTCCGGGAGCTCCGCCT | N/A |
| Primers for Myf5 qPCR-R: | GGCAGCCGTCCGTCATGTCC | N/A |
| Primers for Myod qPCR-F: | TCTGGAGCCCTCCTGGCACC | N/A |
| Primers for Myod qPCR-R: | CGGGAAGGGGGAGAGTGGGG | N/A |
| Primers for Myog qPCR-F: | GAGATCCTGCGCAGCGCCAT | N/A |
| Primers for Myog qPCR-R: | CCCCGCCTCTGTAGCGGAGA | N/A |
| Primers for Pax7 qPCR-F:  AGAGCCCCATCTGTCCTCTC | TTGGGGAACACTCCGCTGTGC | N/A |
| Primers for Pax7 qPCR-R:  ACTGGTAGTCTGCAAAACCAAA | CAGGGCTTGGGAAGGGTTGGC | N/A |
| Primers for Pgc1a qPCR-F:  AGAGCCCCATCTGTCCTCTC | TATGGAGTGACATAGAGTGTGCT | N/A |
| Primers for Pgc1a qPCR-R:  ACTGGTAGTCTGCAAAACCAAA | CCACTTCAATCCACCCAGAAAG | N/A |
| Primers for Ppara qPCR-F:  AGAGCCCCATCTGTCCTCTC | AGAGCCCCATCTGTCCTCTC | N/A |
| Primers for Ppara qPCR-R:  ACTGGTAGTCTGCAAAACCAAA | ACTGGTAGTCTGCAAAACCAAA | N/A |
| Primers for Pparg qPCR-F:  TCGCTGATGCACTGCCTATG | TCGCTGATGCACTGCCTATG | N/A |
| Primers for Pparg qPCR-R:  GAGAGGTCCACAGAGCTGATT | GAGAGGTCCACAGAGCTGATT | N/A |
| Primers for Plin1 qPCR-F | CAAGCACCTCTGACAAGGTTC | N/A |
| Primers for Plin1 qPCR-R | GTTGGCGGCATATTCTGCTG | N/A |
| Primers for Prdm16 qPCR-F: | CCACCAGCGAGGACTTCAC | N/A |
| Primers for Prdm16 qPCR-R: | GGAGGACTCTCGTAGCTCGAA | N/A |
| Primers for Ptch1 qPCR-F: | ACGGTGCTGTGTCCACTCT | N/A |
| Primers for Ptch1 qPCR-R: | CCAGGACGGCAAAGAAGTATC | N/A |
| Primers for Retn qPCR-F: | AAGAACCTTTCATTTCCCCTCCT | N/A |
| Primers for Retn qPCR-R: | GTCCAGCAATTTAAGCCAATGTT | N/A |
| Primers for Sufu qPCR-F: | GTTCTAACCTGAGCGGCGTC | N/A |
| Primers for Sufu qPCR-R: | ATCTGCTCTGTGTCTTTGCCA | N/A |
| Primers for Tcf4 qPCR-F: | GGCGATGAGAACCTGCAAGA | N/A |
| Primers for Tcf4 qPCR-R: | GGTCCTCATCATCGTTATTGCTAGA | N/A |
| Primers for Tnfa qPCR-F: | CCAGACCCTCACACTCA | N/A |
| Primers for Tnfa qPCR-R: | CACTTGGTGGTTTGCTACGAC | N/A |
| Primers for Trim14 qPCR-F: | GTGCGTGTGCAGAAGCTAATC | N/A |
| Primers for Trim14 qPCR-R: | CTGCGTAAACCTTGAGCCTTT | N/A |
| Primers for Ucp1 qPCR-F: | AGGCTTCCAGTACCATTAGGT | N/A |
| Primers for Ucp1 qPCR-R: | CTGAGTGAGGCAAAGCTGATTT | N/A |
| Primers for Zfp423 qPCR-F: | GTCACCAGTGCCCAGGAAGAAGAC | N/A |
| Primers for Zfp423 qPCR-R: | AACATCTGGTTGCACAGTTTACACTCAT | N/A |
| **Software and algorithms** |  |  |
| ImageJ v1.52a | NIH | RRID: SCR_003070 |
| 10x Cell Ranger package | 10x Genomics |  |
| Microsoft Excel | Microsoft | RRID: SCR_016137 |
| GraphPad Prism 8.3.0 | Graphpad | RRID: SCR_002798 |
| Adobe Photoshop (CS6) | Adobe | RRID: SCR_014199 |

**Table S2**

**Single cell RNA-seq information**

**
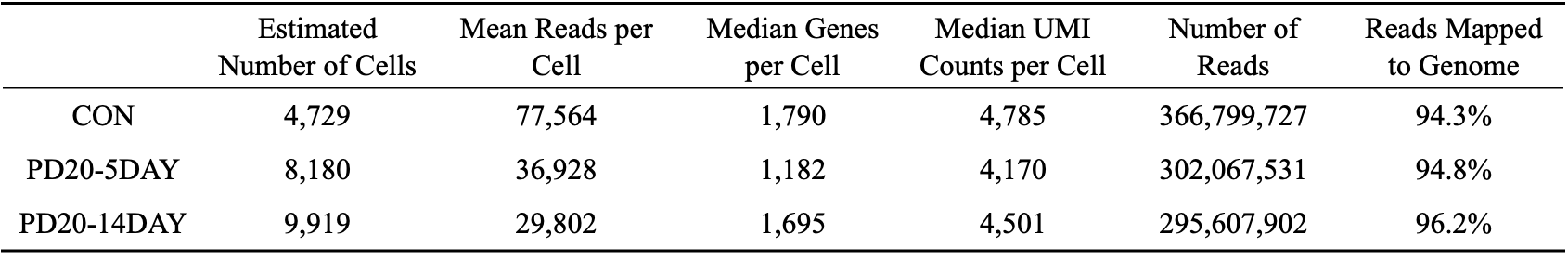
**

**Supplementary methods**

**Differential gene expression, cell type classification, and sub-cluster analysis**

Cell Ranger Single Cell Software Suite v.2.0.1 was used to perform sample demultiplexing, alignment, filtering, and UMI counting. Clustering and gene expression were visualized using the R package Seurat (Version 2.3.4) (*40*). Firstly, cells were filtered to detect >500 genes, and less than 5% of total UMIs were mapped to the mitochondrial genome. Clusters with fewer cells were filtered ahead of downstream analyses. Data were scaled to mitigate the effects of variables: number of genes detected per cell, percentage of mitochondrial reads, and cell cycle stage. Dimensionality reduction was performed using Seurat's t Random Neighbor Embedding method (tSNE). Analysis of differential gene expression (DEGs) between clusters was performed by using the Seurat function FindMarkers with the Wilcoxon test. Cell types were determined using a combination of marker genes identified from the literature and a gene ontology of cell types. Violin plots, heatmaps, and individual tSNE plots for a given gene were generated using the Seurat toolkit VlnPlot, DoHeatmap, and FeaturePlot functions, respectively. To perform subclustering, we used Seurat’s subset function to extract the cell types of interest (FAPs and myeloid-derived cells). We performed subsequent dimensionality reduction, clustering, and tSNE visualization in Seurat.

**Pseudotemporal analysis**

Pseudotemporal analysis was performed on filtered cells using the R package monocle Version 2.4 with default settings. Pseudotime sorting was performed using the function &quot; reduce dimension&quot; with max_components set to 2 and reduction_method set to DDRTree. Next, significantly affected genes were obtained from the top 50 markers in the cluster by using the function differsGeneTest(fullModelFormulaStr = ~Pseudotime) and plotted using the function plot_pseudotime_heatmap. num_cluster was set to 4 to obtain modules of four significantly changed genes with similar trends according to their pseudo-temporal expression patterns.

**Protein extraction and western blot**

Total protein was isolated from cells or tissues using RIPA buffer (25 mM Tris HCl pH7.6, 150 mM NaCl, 1% NP-40, 1% sodium deoxycholate, 0.1% SDS) supplemented with protease and phosphatase inhibitor cocktails (Thermo Fisher Scientific). Protein separation and western blot analysis were conducted as described. Specific protein bands were densitometrically quantified using ImageJ software (v 1.52q).

**Total RNA extraction and qRT-PCR**

Total RNA was extracted from cells or tissues using Trizol Reagent following the manufacturer’s instructions. The purity and concentration of total RNA were measured by a spectrophotometer (Nanodrop 2000, Thermo Fisher Scientific) at 260 and 280 nm. Absorption rates (260/280 nm) of all samples ranged between 1.8 and 2.0. First-strand cDNA was synthesized using random primers with a reverse transcription kit. Real-time PCR was carried out with a Roche Lightcycler 480 PCR System using SYBR Green Master Mix and gene-specific primers. The 2−ΔΔCT method was used to analyze the relative changes in gene expression normalized against 18S rRNA as an internal control.
